# A High-Content Screening Approach to Identify MicroRNAs Against Head and Neck Cancer Cell Survival and EMT in an Inflammatory Microenvironment

**DOI:** 10.3389/fonc.2019.01100

**Published:** 2019-11-08

**Authors:** Bruno Sangiorgi, Felipe Canto de Souza, Ildercílio Mota de Souza Lima, Josiane Lilian dos Santos Schiavinato, Amanda Cristina Corveloni, Carolina Hassibe Thomé, Wilson Araújo Silva, Vitor Marcel Faça, Dimas Tadeu Covas, Marco Antônio Zago, Rodrigo Alexandre Panepucci

**Affiliations:** ^1^Center for Cell-Based Therapy (CTC), Regional Blood Center of Ribeirão Preto, Ribeirão Preto, Brazil; ^2^Department of Genetics and Internal Medicine, Ribeirão Preto Medical School, University of São Paulo (FMRP-USP), Ribeirão Preto, Brazil; ^3^Department of Biochemistry and Immunology, Ribeirão Preto Medical School, University of São Paulo (FMRP-USP), Ribeirão Preto, Brazil

**Keywords:** head and neck squamous cell carcinoma, high-content screening, microRNAs, epithelial-mesenchymal-transition, inflammation, NF-κB

## Abstract

Head and neck squamous cell carcinoma (HNSCC) is among the most common cancer types. Metastasis, the main cause of death by cancer, can be promoted by an inflammatory microenvironment, which induces epithelial-mesenchymal transition (EMT) through a NF-κB-mediated stabilization of Snail. Here, we aimed to explore how microRNAs (miRs) can affect cell survival and EMT in HNSCC cells under an inflammatory microenvironment. By using a high-content screening (HCS) approach, we evaluated alterations in morphometric parameters, as well as expression/localization of Snail/Slug, in HNSCC cells primed with TNF-α. Based on those quantitation, we established the optimal experimental conditions of EMT induction driven by TNF-α. Those conditions were applied to cells transfected with distinct miRs (*N* = 31), followed by clusterization of miRs based on alterations related to cell survival and EMT. The signaling pathways enriched with molecular targets from each group of miRs were identified by *in silico* analyses. Finally, cells were transfected with siRNAs against signaling pathways targeted by miRs with anti-survival/EMT effect and evaluated for alterations in cell survival and EMT. Overall, we observed that TNF-α, at 20 ng/ml, induced EMT-related changes in cell morphology, Snail/Slug expression, and cell migration. Predicted targets of miRs with anti-survival/EMT effect were enriched with targets of NF-κB, PI3K/ATK, and Wnt/beta catenin pathways. Strikingly, individual gene silencing of elements from those pathways, namely *RELA* (NF-kB), *AKT1* (PI3K/AKT), and *CTNNB1* (Wnt/beta catenin) reduced cell survival and/or expression of Snail/Slug in cells stimulated with TNF-α. As a whole, our HCS approach allowed for the identification of miRs capable of inhibiting cell survival and EMT considering the presence of an inflammatory microenvironment, also indicating the common signaling pathways and molecular targets most likely to underlie those alterations. These findings may contribute to the development of targeted therapies against HNSCC.

## Introduction

Head and neck squamous cell carcinoma (HNSCC) comprises a group of upper aerodigestive tract neoplasia and is among the ten types of cancer with the highest incidence and mortality in the world (1). Over the past decades, despite advances in treatment strategies of HNSCC, it was observed a growth in mortality associated with distant metastases (2). Studies to date demonstrated that metastasis initiation is promoted by tumor cells that undergoes epithelial-mesenchymal transition (EMT), a transformation process which cells acquire a mesenchymal-like phenotype and dislodges from the tumor bulk, invading adjacent vessels and entering in the circulation (3).

EMT events are coordinated by transcription factors known as “EMT master regulators,” including members of the Snail family: SNAI1 (Snail) and SNAI2 (Slug), which are capable of both silencing and promoting the expression of genes related to epithelial and mesenchymal phenotypes, respectively (4). As a consequence of the “EMT master regulators” activity, cancer cells undergo drastic phenotypic changes in cell morphology: from polygonal to elongated, expression of cell adhesion proteins: downregulation of E-Cadherin and upregulation of N-Cadherin and integrins, expression of structural proteins: upregulation of Vimentin, among others that lead to the formation of mesenchymal cancer cells with migratory/invasive capacities (5).

Increasing literature data have established that, for several types of cancer including HNSCC, the presence of an inflamed tumor microenvironment is associated with tumor progression, the acquisition of EMT-like features by cancer cells and the formation of metastasis (6). In different types of cancer, multiple lines of evidence have supported that inflammatory cytokines secreted by tumor-associated macrophages (which can represent half of the tumor mass), including tumor necrosis factor alpha (TNF-α), are capable of inducing EMT events in cancer cells (7). TNF-α activates the nuclear factor kappa b (NF-κB) signaling pathway, which the main effector p50/p65 (RelA) promotes the nuclear translocation of Snail, thereby inducing EMT (8). Additionally, NF-κB crosstalk with other oncogenic signaling pathways in HNSCC including Ras/MAPK, PI3K/AKT, and Wnt/beta catenin, that collectively promotes cancer cell survival, evasion from apoptosis and therapy resistance (9, 10). Due to the complexity of intracellular signaling pathways and tumor microenvironment in cancer, including HNSCC, a multi-target therapy (targeting multiple signaling pathways) may be an interesting therapeutic approach (11).

MicroRNAs (miRs) are a class of small non-coding RNAs that act predominantly through the destabilization and degradation of multiple targeted messenger RNAs (mRNAs) thereby affecting several biological processes independently (12, 13). In HNSCC, as in other types of cancer, there is mounting evidence that miRs are capable of interfering in multiple cellular processes, such as cancer cell proliferation, invasion, and apoptosis, thereby promoting (oncomiRs) or inhibiting (tumor suppressor miRs) the progression from normal tissue to carcinoma and subsequently metastasis (14, 15). Importantly, the HNSCC oncomiR: miR-21 and tumor suppressor miR: miR-29, are both involved in transcriptional networks that regulates the activity of the NF-κB signaling pathway (14, 16), highlighting the importance of NF-κB as a regulator of both inflammation and tumor progression in HNSCC.

Since its discovery, miRs have been drawing attention due to their capacity to be used either as prognostic biomarkers or in miR-based targeted therapies against cancer (17, 18). Currently, miR-based targeted therapeutic strategies comprehends the delivery of either mimetics of miRs with tumor suppressor activity (microRNA replacement or restoration therapy) or molecules capable of inactivating oncomiRs (microRNA reduction or inhibition therapy) (19). Importantly, a functional study conducted by Lindenbergh-van der Plas and coworkers provided a proof-of-concept that miRs can be used to selectively kill HNSCC cancer cells (20). However, despite the potential use of miRs in drug discovery and therapeutic applications, it is a current challenge to identify, among the several signaling pathways regulated by a given miR, those that has an effective therapeutic value (21).

In the last decade, advances in the High-Content Screening (HCS) approach (cell-based functional screens based on automated microscopy and image analysis) allowed for the quantitative measurement of a broad spectrum of phenotypic alterations at a cellular level (22). Noteworthy, the advantage of the HCS approach to measure the phenotype in a multiparametric fashion makes it especially suited to investigate the pleiotropic effects exerted by miRs (23). In addition, target-prediction tools can also be utilized for the identification of the molecular targets shared by groups of miRs and thereby indicating the ones that are most likely responsible for the observed effects (24). In the present work, through an HCS approach and *in-silico* analysis, we investigated the capacity of miRs to alter the phenotypic features related to tumor progression (e.g., cell survival) and metastasis (e.g., EMT) in HNSCC cells considering the presence of an inflammatory microenvironment. Overall, we have identified miRs capable of inhibiting cell survival and EMT as well as potential targets and signaling pathways involved in the observed effects.

## Materials and Methods

### Study Design

**Figure 1:**
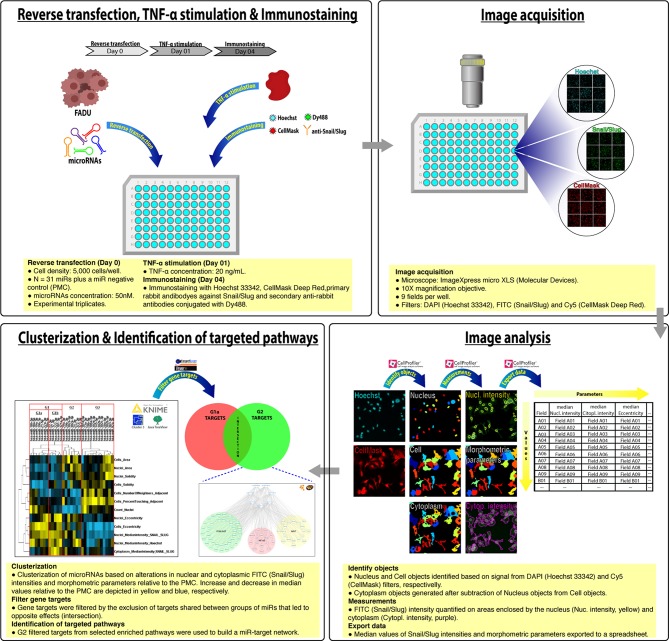
Study design. Reverse transfection, TNF-α stimulation and Immunostaining: Cells from the FADU cell line were transfected (day 0) with microRNAs (*N* = 31), followed by stimulation with TNF-α (20 ng/mL, Day 01) and immunostaining with primary rabbit antibodies against Snail/Slug, secondary anti-rabbit antibodies conjugated with Dy488, nuclear (Hoechst) and cytoplasmic (CellMask) fluorescent dyes (Day 04). Image acquisition: Images (9 fields per well) were acquired using a 10X objective and excitation/emission filters DAPI (Hoechst), FITC (Snail/Slug), and Cy5 (CellMask), using an ImageXpress Micro XLS HCS system (Molecular Devices). Image analysis: Nuclei and corresponding cytoplasm objects were identified and segmented based on images from DAPI (Hoechst) and Cy5 (CellMask) channels, respectively. FITC (Snail/Slug) intensity on nuclei and cytoplasm, as well as morphometric parameters were then quantified. Median values per field were exported into a spreadsheet. Clusterization and Identification of targeted pathways: Based on alterations in median values per well relative to the PMC, microRNAs were subjected to an unsupervised hierarchical clustering. After the exclusion of genes commonly targeted by miRs from G1a (pro-survival/EMT) and G2 (anti-survival/EMT), genes from signaling pathways targeted by G2 miRs were used to generate a microRNA regulatory network.

The design of this study is illustrated in Figure 1. Cells from the FADU cell line were transfected (reverse transfection) into 96 well plates with miR mimetics (*N* = 31 plus a miR negative control) in experimental triplicates, followed by stimulation with TNF-α (20 ng/mL) for 72 h and immunostaining with primary rabbit antibodies against Snail/Slug, secondary anti-rabbit antibodies conjugated with Dy488, nuclear (Hoechst) and cytoplasmic (CellMask) fluorescent dyes. Images (nine fields per well) were acquired using a 10X objective and excitation/emission filters DAPI (Hoechst), FITC (Snail/Slug), and Cy5 (CellMask), using an ImageXpress Micro XLS HCS system (Molecular Devices). With aid of CellProfiler, images from filters DAPI (Hoechst) and Cy5 (CellMask) were used to identify nuclear, cell and cytoplasm objects, followed by quantification of nuclear and cytoplasmic median FITC (Snail/Slug) intensity, as well as morphometric parameters. Median values per field were exported into spreadsheets and with help of KNIME software, we obtained the percentage change of the median values per well relative to the miR negative control (PMC). By using Cluster3 and Java TreeView software, we performed a unsupervised hierarchical clustering of miRs by which the four groups of miRs (G1a, G1b, G2, and G4) were identified. With help of KNIME and Targetscan software, we identified the genes targeted by most (N-2, minimum of 4) of the microRNAs in each group. With help of Venny online tool, genes targeted by groups that led to opposite phenotypic effects were identified and excluded from further analyses. With aid of Database for Annotation, Visualization and Integrated Discovery (DAVID, version 6.7) online tool, we identified signaling pathways enriched with filtered targets. With help of the Kyoto Encyclopedia of Genes and Genomes (KEGG) database, the filtered targets from G2 miRs were assigned to the NF-kB, PI3K/AKT, and Wnt/beta-catenin signaling pathways, which were used to generate a microRNA regulatory network with help of Cytoscape software. Based on information from those analyses, secondary functional assays using siRNAs were designed to evaluate the effect, in cell survival and EMT, of interferences in NF-kB, PI3K/AKT, and Wnt/beta-catenin signaling pathways.

### Cell Lines

Cells derived from the HNSCC cell lines FADU (oropharynx), HN30 (pharynx), and UMSCC1 (floor of mouth) were cultured in Dulbecco's modified Eagle's medium (DMEM) supplemented with 10% fetal bovine serum (FBS), 50 U/mL penicillin and 50 μg/mL streptomycin. Cells were passaged by using a 10% trypsin solution.

### Reagents

Throughout this work, cells were treated with TNF-α (300-01A, PrepoTech, USA) or mitomycin C (MMC, M4287, sigma-aldrich, USA). For immunostaining, we used the nuclear dye Hoechst 33342 (10 μg/mL; H1399, Thermo Scientific, USA) and cytoplasmic dye HCS CellMask Deep Red (5 μg/mL, H10294, Thermo Scientific, USA), primary antibodies: Anti-N-Cadherin mouse IgG2ab mAb (SC-271386, Santa Cruz Biotechnology, USA), Rabbit anti-Snail/Slug (ab180714, abcam, USA), Goat anti-Vimentin (sc-7558, Santa Cruz Biotechnology) and Mouse-anti-Caspase-7 (cleaved caspase-7 p10, clone h207, sc-22179, Santa Cruz Biotechnology, USA), as well as secondary antibodies: Goat anti-Rabbit DyLight 488 (dy488, 35553, Thermo Scientific, USA), Goat anti-Rabbit DyLight 594 (35561, Thermo Scientific, USA) DyLight 488 mouse (35503, Thermo Scientific, USA) and Donkey anti-Goat DyLight 594 (SA5-1088, Thermo Scientific, USA). For western blot, we used the antibodies rabbit anti-snail (#3879, Cell Signaling, USA), rabbit anti-slug (#9585, Cell Signaling, USA), rabbit anti-vimentin (#5741, Cell Signaling, USA), rabbit anti N-cadherin (#13116, Cell Signaling, USA), rabbit anti-β-catenin (#8480, Cell Signaling, USA) and mouse anti-β-actin (sc-81178, Santa Cruz, CA).

### Western Blot

For protein extraction and quantification, cells were washed with PBS and disrupted in lyses buffer (20 mM Tris-HCl, 150 mM NaCl, 1 mM Na2EDTA, 1 mM EGTA, 1% Triton X-100, 2.5 mM sodium pyrophosphate, 1 mM β-glycerophosphate, 1 mM Na3VO4 and 1 μg/ml leupeptin). After three sonication cycles at 45 W for 5 min each in a sonicator bath, the samples were centrifuged at 20,000 × g for 30 min at 4°C. The protein concentration was determined by the Bradford method (Bio-Rad, Hercules, CA).

Proteins were submitted to SDS–PAGE and electrotransferred to PVDF membranes (GE Lifesciences, Pittsburgh, PA, USA). Membranes were blocked with 5% non-fat dry milk in 0.1% Tween-TBS and incubated with the primary antibody. After 1 h of incubation with horseradish peroxidase-conjugated goat anti-rabbit IgG (#7074, Cell Signaling) or horse anti-mouse IgG (#7076, Cell Signaling) secondary antibodies The antibody-protein complex was detected using ECL Western Blotting Detection Reagents (GE Lifesciences) using a CCD-Camera (Image QuantLAS 4000 mini, Uppsala, Sweden). Densitometric analysis was performed using the ImageJ software, and bands were normalized to the constitutive protein β-actin.

### MicroRNA Mimics and siRNAs

Transfection assays were carried out with human microRNA mimetic molecules (50 nM, Thermo Scientific) or synthetic siRNA molecules (10 nM; Supplementary File 1).

### Reverse Transfection

Reverse transfection assays were performed using lipofectamine LTX transfection reagent (15338100, Thermo Scientific) and synthetic miRs/siRNAs according to manufacturer's instructions. Transfection efficiency was calculated by evaluating the percentage reduction in cell numbers following transfection with a cytotoxic siRNA against Ubiquitin (siUBC) as compared to cells transfected with a control miR (PMC) or siRNA (siCTR).

### Immunostaining

Cells were fixed and permeabilized with a 2% formaldehyde solution in methanol for 20 min at −20°C. Quenching of formaldehyde was achieved by incubation for 15 min with a 0.1 M glycine solution and blocking with a 1% FBS solution for 30 min. Afterwards, cells were incubated for 1 h at room temperature with primary antibodies, followed by incubation for 45 min with a solution containing secondary antibodies and nuclear/cytoplasmic dyes.

### Image Analysis

Image analyses were performed with aid of MetaXpress software (Molecular Devices, USA) or CellProfiler (version 2.2.0, Broad Institute, USA). Briefly, images from functional assays that aimed to evaluate only the presence/absence of fluorescent dyes or markers were analyzed using MetaXpress software, whereas CellProfiler was used to analyze images from functional assays aiming both the evaluation of morphometric parameters and the presence and subcellular localization of fluorescent markers. Data from image analyses were processed with the aid of KNIME software (version 3.2.0).

### HCS-Based Functional Assays

Alterations in cell morphology and expression/localization of proteins were assessed through HCS-based functional assays, which comprises of:

- MiR/siRNA reverse transfection of FADU cells into 96-well culture plates (CLS3603, Corning, USA);
- Stimulation or not with TNF-α, 24 h after reverse transfection;
- Immunostaining using antibodies, nuclear and cytoplasmic dyes;
- Image acquisition (9 fields per well) with aid of an ImageXpress^®^ Micro XLS High-Content Screening (HCS) system (Molecular Devices, USA), using a 10X magnification objective and excitation/emission filters DAPI, FITC, Cy3, Texas Red, and Cy5;
- Image analysis with aid of MetaXpress (Molecular Devices, USA) or CellProfiler (version 2.2.0, Broad Institute, USA) software.

### Migration Assay

Cells were seeded on culture plates specific for migration assays Oris Pro Cell Migration Assay, 96 wells (PROCMA5, Platypus Technologies, USA). After 24 h, cells were treated with 0.2 μg/mL of mitomycin C for 2 h (to suppress proliferation), followed or not by incubation with TNF-a at 20 or 50 ng/mL for 72 h (experimental triplicates). Images were acquired using a 4X phase-contrast objective after cell seeding and at the endpoint using the ImageXpress HCS system. The area occupied by cells was quantified after cell seeding and at the endpoint, which were used to measure cell migration using *M* = *(Ae/As* ^*^ *100)-100*, in which *M* = migration, *Ae* = Area occupied by cells at the endpoint, *As* = Area occupied by cells 24 h after seeding.

### Clusterization of miRs

A unsupervised hierarchical clustering of miRs was performed with aid of Cluster 3 software (25) and visualized with help of Java TreeView software (26). Groups of miRs were classified as of pro/anti survival/EMT properties based on alterations in the following phenotypic parameters: “count nuclei (cell survival),” “cells eccentricity (EMT),” “Nuclei Median Intensity Snail/Slug (EMT),” and “Cytoplasm Median Intensity Snail/Slug (EMT).”

### Identification of Genes and Signaling Pathways Targeted by Groups of miRs

Using the KNIME software (version 3.7) and TargetScan database of predicted miR targets (version 7.1) (27), we created a pipeline to identify the transcripts commonly targeted by most of the miRs contained in each of the identified groups (N-2, minimum of 4). Venn diagrams were generated using Venny 2.1 online tool (bioinfogp.cnb.csic.es/tools/venny) by comparing the identified targets from groups of miRs with opposite phenotypic effects, followed by the exclusion (filtering) of the shared targets. Afterwards, with help of the Database for Annotation, Visualization and Integrated Discovery (DAVID, version 6.7) (28), we identified signaling pathways that were enriched with the filtered targets from each group of miRs.

The filtered targets were assigned to their given signaling pathways according to information available on the Kyoto Encyclopedia of Genes and Genomes (KEGG) database about the following signaling pathways: NF-κB (hsa04064), PI3K/AKT (hsa04151), and Wnt (hsa04310) (29). The miRs and targets from the selected pathways were used to generate a microRNA regulatory network with aid of Cytoscape software (30).

### Quantitative PCR (qPCR)

RNA extraction was performed using TRIZOL reagent (Invitrogen Life Technologies, Grand Island, NY, USA) and total RNA was reverse transcribed using the High Capacity cDNA Reverse Transcription Kit (Applied Biosystems, Foster City, CA, USA), according to the manufacturer's instructions. Gene expression qPCR reactions were carried in duplicates with Power SYBR Green Master Mix (Applied Biosystems) and primers for AKT2 (Forward: AAGGATGAAGTCGCTCACAC; Reverse: ACTCCATCACAAAGCACAGG), CCND1 (Forward: CCCGCACGATTTCATTGAAC; Reverse: GGCGGATTGGAAATGAACTTC), GAPDH (Forward: GAAGGTGAAGGTCGGAGTC; Reverse: GAAGATGGTGATGGGATTTC); IL6 (Forward: ATGCAATAACCACCCCTGAC; Reverse: GAGGTGCCCATGCTACATTT); MYC (Forward: CAGATCAGCAACAACCGAAA; Reverse: GGCCTTTTCATTGTTTTCCA) and RELA (Forward: TGACAAGGTGCAGAAAGAGG; Reverse: CACATCAGCTTGCGAAAAGG) using a CFX96 Real-Time PCR system (Bio-Rad). Relative gene expression levels were assessed using the 2^−ΔΔCt^ strategy (31).

### Statistics

All statistical analyses were performed with aid of GraphPad Prism software version 5.0. Comparisons between multiple experimental conditions were performed using either unpaired *t*-test or univariate “ONE-WAY Anova” test. Statistical significance was considered at *p* < 0.05.

## Results

### Stimulation With Tumor Necrosis Factor Alpha Leads to EMT-Related Morphometric Alterations

**Figure 2:**
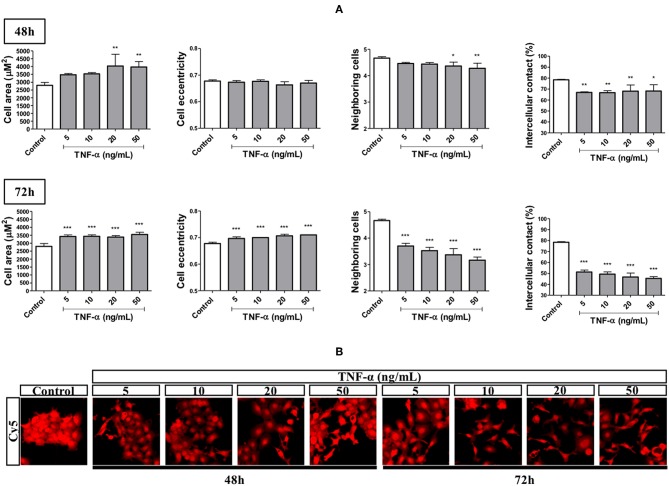
Effects of TNF-α in EMT-related morphometric parameters. The FADU cell line was stimulated or not (Control) with TNF-α (5 to 50 ng/mL), for 48 or 72 h (squares). At the endpoint, the cells were stained with nuclear (Hoechst) and cytoplasmic (CellMask) fluorescent dyes. Images (nine fields per well) were acquired using a 10X objective and excitation/emission filters DAPI (Hoechst) and Cy5 (CellMask), using an ImageXpress Micro XLS HCS system (Molecular Devices). Images were analyzed using CellProfiler, in order to quantify the following parameters: cell area, cell eccentricity, number of neighboring cells and percentage of intercellular contact. **(A)** Quantitative changes (Mean +-SD) in morphometric parameters due to TNF-α stimulation for 48 and 72 h. **(B)** Representative images of cells stimulated with TNF-α for 48 and 72 h. Statistically significant differences (ONE-WAY Anova with Tukey post-test), in comparison to the control condition: ^*^p < 0.05; ^**^p < 0.01; ^***^p < 0.001.

We performed an HCS-based functional assay in cells primed with TNF-α at different concentrations and time points followed by quantification of morphometric features. Stimulation for 48 h with TNF-α, at all concentrations used (5–50 ng/mL), reduced the percentage of intercellular contact by around 15% while not changing cell eccentricity (elongation). On this same endpoint, we found a trend for increase and decrease in cell area and number of neighboring cells, respectively, in a concentration-dependent manner, attaining significance at 20 and 50 ng/mL. On the other hand, stimulation for 72 h with all TNF-α concentrations used, led to a significant increase in cell area (around 1,000 μm^2^) and cell eccentricity. These changes were accompanied by significant reductions in both the number of neighboring cells (by around 1.5) and the percentage of intercellular contact (by around 30%; Figure 2).

### Stimulation With Tumor Necrosis Factor Alpha Leads to the Expression of EMT-Related Proteins

An HCS-based functional assay was done in cells primed with TNF-α for 72 h and at different concentrations, with further quantification of changes in the percentage of cells positive for markers of EMT. Generally, we observed that stimulation with TNF-α led to a concentration-dependent increase in the percentage of cells positive for all markers evaluated. More specifically, the higher concentration of 50 ng/mL led to a significant increase in the percentage of cells expressing N-Cadherin in the cytoplasm (from 30 to 50%, approximately). Moreover, the percentage of cells expressing cytoplasmic Vimentin significantly increased by 25% after treatment with TNF-α at 20 and 50 ng/mL. Finally, the percentage of cells expressing Snail/Slug in the nucleus significantly increased at all concentrations of TNF-α, ranging from below 20% (in untreated cells) up to above 60% ate the highest TNF-α concentration.

**Figure 3:**
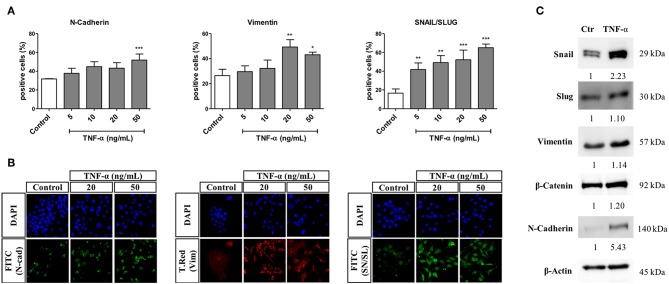
Effects of TNF-α in the expression of EMT-related proteins. The FADU cell line was stimulated or not (Control) with TNF-α (5 to 50 ng/mL), for 72 h, followed by analyses of EMT-related protein levels by immunostaining or western blot. After immunostaining using antibodies against N-cadherin (N-cad), Vimentin (Vim) and Snail/Slug (SN/SL), cells were co-stained with nuclear (Hoechst) and cytoplasmic (CellMask) fluorescent dyes. Images (nine fields per well) were acquired using a 10X objective and excitation/emission filters DAPI (Hoechst), FITC (N-Cadherin and SN/SL), and Texas Red (T.Red; Vimentin), using an ImageXpress Micro XLS HCS system (Molecular Devices). Images were analyzed using MetaXpress, in order to evaluate the percentage of cells positive for each protein in the nucleus or cytoplasm. For western blot, protein detection was achieved using antibodies against Snail, Slug, Vimentin, beta-catenin and N-Cadherin. **(A)** Nuclear or cytoplasmic quantitative changes (Mean +-SD) in protein levels of N-Cadherin, Vimentin and Snail/Slug, as evaluated by immunofluorescence. **(B)** Representative images of stained cells stimulated with TNF-α at 20 and 50 ng/mL. **(C)** Alterations (fold change) in the protein levels of Snail, Slug, Vimentin, beta-catenin and N-Cadherin, as evaluated by western blot after 72 h of TNF-α stimulation (20 ng/mL). Statistically significant differences (ONE-WAY Anova with Tukey post-test), in comparison to the control condition: ^*^p < 0.05; ^**^p < 0.01; ^***^p < 0.001.

By western blot, we observed that after 72 h of treatment with TNF-α at 20 ng/mL, the protein levels of Snail and N-Cadherin increased by 2.2- and 5.4- fold in comparison to the untreated control group, respectively. Moreover, the levels of Slug and Vimentin were increased by around 1.1-fold, whereas the protein level of beta-catenin was increased by 1.2-fold (Figure 3).

### Tumor Necrosis Factor Alpha Stimulation Induces Cell Migration

**Figure 4:**
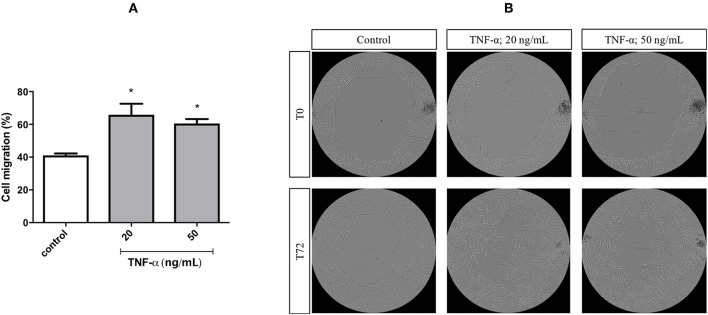
Cell migration of FADU cells following stimulation with TNF-α. The FADU cell line was cultured for 24 h in cell culture plates designed for migration assays, treated with mitomycin for 2 h (to inhibit cell proliferation) and stimulated or not (Control) with TNF-α at 20 or 50 ng/mL for 72 h. Phase-contrast transmitted light images were acquired, with an ImageXpress Micro XLS HCS system (Molecular Devices), using a 4X objective. With aid of CellProfiler, cell migration was quantified based on the percentage of increase in the area occupied by cells. **(A)** Percentage of cell migration after 72 h (Mean +-SD). **(B)** Representative images of different experimental conditions at initial time (T0) and after 72 h (T72). Statistically significant differences (ONE-WAY Anova with Tukey post-test), in comparison to the control condition: ^*^p < 0.05.

A migration assay was performed in cells treated with mitomycin C and primed with TNF-α at different concentrations, for 72 h. Stimulation with TNF-α at 20 and 50 ng/mL increased the migratory capacity of FADU cells by around 30 and 20%, respectively (Figure 4).

### HCS-Based miR Screening Identifies miRs With Distinct Effects on Cell Survival and EMT

An HCS-based functional assay was performed in cells transfected with our library of miRs (*N* = 31), followed by priming with TNF-α (20 ng/mL) for 72 h, in order to evaluate changes on morphometric parameters and Snail/Slug levels/localization. We identified miRs that altered cell survival (nuclei count) and EMT-related features including nuclear Snail/Slug levels and morphometric parameters such as cellular/nuclear area, eccentricity and cell distancing relative to the miR negative control. After unsupervised hierarchical clustering, miRs were distributed into three main groups (G1, G2, and G3). Based on distinct alterations in cell survival (nuclei count), G1 was further subdivided into the subgroups G1a and G1b.

MiRs from G3 led to a pro-survival and anti-EMT effect, as seemed by increase in cell survival and epithelial phenotype: tightly packed juxtaposed (higher number of neighboring cells and percentage of touching) round cells (lower eccentricity and higher solidity) with low nuclear Snail/Slug levels. In contrast, the subgroup G1b had the exact opposite phenotypic features (anti-survival and pro-EMT), with low cell counts, high Snail/Slug intensity and interspersed cells with mesenchymal phenotype (high eccentricity and low solidity). Since a higher cell density by itself excerpts a strong inhibitory effect on EMT, while a lower cell density is able to promote it (32), G1b and G3 are hereafter referred as anti-survival and pro-survival miR groups, respectively.

**Figure 5:**
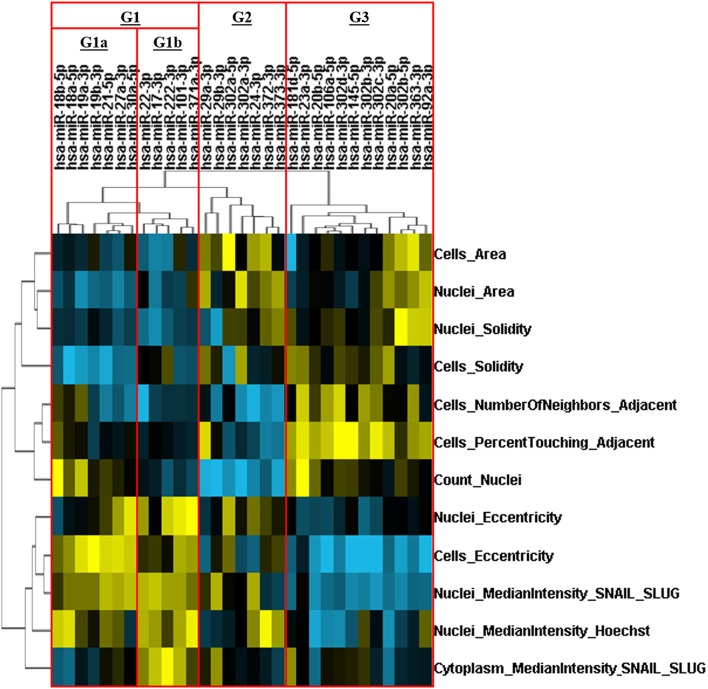
Hierarchical clustering of miRs based on their effects on multiparametric phenotypic alterations. The FADU cell line was transfected with human miRs mimics, for 24 h, followed by stimulation with TNF-α (20 ng/mL) for 72 h. After immunostaining with antibodies against Snail/Slug (SN/SL) and co-staining with nuclear (Hoechst) and cytoplasmic (CellMask) dyes, images were acquired with an ImageXpress Micro XLS HCS system (Molecular Devices), using a 10X objective and excitation/emission filters DAPI (Hoechst), FITC (SN/SL), and Cy5 (CellMask Deep Red). With aid of CellProfiler, we evaluated several morphometric parameters, besides cell quantity (count nuclei) and the presence/location of Snail/Slug. Multiparametric phenotypic profiles, describing the effects of each miR, were obtained and subjected to an unsupervised hierarchical cluster analysis. Heatmap showing the multiparametric phenotypic profiles induced by each miR, and the four groups of miRs identified (G1a, G1b, G2, and G3. Red rectangles). Increase and decrease relative to PMC are depicted in yellow and blue, respectively.

The subgroup G1a promoted cell survival while increasing nuclear Snail/Slug levels and morphological EMT-related features comparable to that of G1b; thus, clearly displaying a pro-survival/EMT effect. Otherwise, miRs from G2 had the strongest negative impact on survival while some of its members were still capable of reducing or preventing the increase of cell eccentricity or nuclear Snail/Slug levels, thereby displaying a anti-survival/EMT effect (Figure 5).

### Anti-survival/EMT miRs Target Inflammatory-Associated Pathways

**Figure 6:**
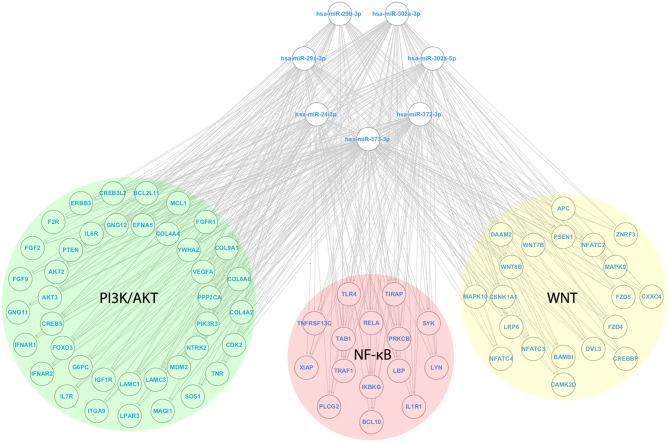
MicroRNA Regulatory network with miRs from the anti-survival/EMT group. A microRNA regulatory network was composed of miRs from the anti-survival/EMT group, along with their targets from the PI3K/AKT (Green circle), NF-κB (Red circle), and Wnt (Yellow circle) signaling pathways. For each pathway, targets from the outer portion of the circle are the ones targeted by no more than 5 miRs, whereas the ones from the inside portion of the circle are the ones targeted by more than 5 miRs (maximum of 7). The following genes are shared between the signaling pathways NF-κB and PI3K/AKT: *IKBKG, RELA, SYK*, and *TLR4*; NF-κB and WNT: *PRKCB*.

After identifying the genes collectively targeted by the miRs from G1a, G1b, G2, and G3, we eliminated targets shared by groups that led to opposite phenotypic effects. Thereby, targets shared between G1a (pro-survival/EMT) and G2 (anti-survival/EMT), as well as G1b (anti-survival) and G3 (pro-survival) were eliminated from further analyses as they were considered not relevant for the phenotypic effects driven by the groups of miRs (Supplementary File 2). Then, the filtered targets were used for enrichment analysis on signaling pathways and biological processes (Supplementary File 3). Strikingly, we found that miRs from both G1b (anti-survival) and G2 (anti-survival/EMT) targeted inflammatory pathways including “TNF signaling pathway (G1b)” and “Toll-like receptor signaling pathway (G2)” and shared targets from the following pathways: NF-κB (*IKBKG);* PI3K/AKT (*AKT2*), and MAPK (*MAPK9*). Moreover, we found that miRs from G2 also targeted additional genes (not found in G1b) from the NF-κB and PI3K/AKT pathways including *RELA* and *PIK3R3*, respectively. Finally, the Wnt/beta catenin signaling pathway was also found to be enriched with targets from G2. The miRs from G2 (anti-survival/EMT) and its targets from the NF-κB, PI3K/AKT, and Wnt/beta-catenin signaling pathways were used to generate a microRNA regulatory network (Figure 6).

### Anti-survival/EMT miRs Reduce the Transcript Levels of Their Direct and Indirect Targets

**Figure 7:**
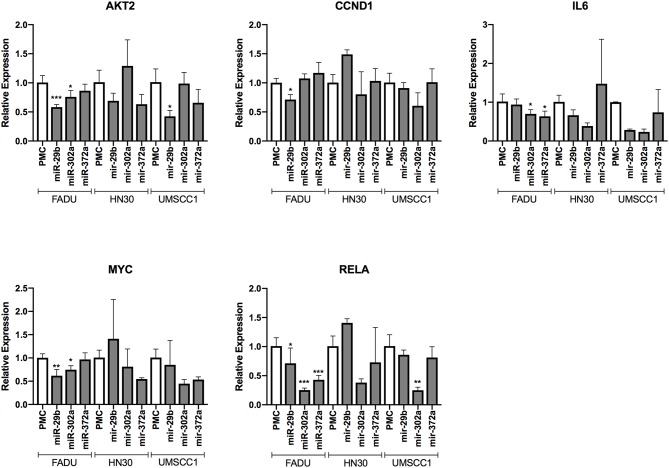
Changes in the transcript levels of direct and indirect targets of anti-survival/EMT miR group following miR transfection. The cell lines FADU, HN30, and UMSCC1 were transfected with PMC (control) or miR mimics from G2 (miR-29b-3p, miR-302a-3p, and miR-372), followed by TNF-α stimulation (20 ng/mL) for 48 h and qPCR with primers for *AKT2, CCND1* (cyclin D1), *IL6, MYC*, and *RELA*. Alterations in relative gene expression levels, relative to cells transfected with miR-CTR (PMC). Statistically significant differences (*t*-test), in comparison to the reference control group (PMC): ^*^p < 0.05; ^**^p < 0.01; ^***^p < 0.001.

We evaluated the capacity of three miRs with anti-survival/EMT effects (miR-29b-3p, miR-302a-3p, and miR-372-3p) to reduce the transcript levels of direct predicted targets, as well as indirect downstream transcriptional targets. Overall, with the exception of *CCND1* (an indirect target of the miRs), the targets were downregulated by most of the miRs among the cell lines, however a stronger effect and less variability were observed on the FADU cell line. More specifically, we observed a reduction on the expression levels of the direct target *AKT2*, with the exception of miR-372 (on FADU) and miR-302a (on HN30 and UMSCC1). The same was observed for the direct target *RELA*, with the exception of miR-29b (on HN30 and UMSCC1) and miR-372 (on HN30 and UMSCC1). As for the indirect targets, *MYC* was downregulated with the exception of miR-372 (on FADU) and miR-29b (on HN30 and UMSCC1). Finally, *IL6* was also downregulated by the miRs, with the exception of miR-29b (on FADU), and miR-372 (on HN30 and UMSCC1) (Figure 7).

### Interferences in Signaling Pathways Targeted by Anti-survival/EMT miRs Partially Recapitulate Their Effects

HCS-based functional assays were done in cells transfected with siRNAs against elements of signaling pathways regulated by the anti-survival/EMT miRs: *RELA* (siRELA, NF-κB pathway), *AKT1* (siAKT1, PI3K/AKT pathway), and *CTNNB1* (siCTNNB1, Wnt/beta-catenin signaling pathway), besides a non-targeting control siRNA (siCTR) and cytotoxic siRNA (siUBC), followed or not by stimulation with TNF-α.

After 72 h of TNF-α stimulation, siRNA-mediated knockdown of *RELA* transcripts led to an expressive reduction in cell number (count nuclei) and number of neighboring cells, while increased cell eccentricity. Silencing the expression of *AKT1* (of high homology with *AKT2*, target of G2) led to a discrete reduction in cell number, while increased cell area and significantly reduced nuclear and cytoplasmic levels of Snail/Slug. Finally, knockdown of *CTNNB1* significantly reduced cell number, cytoplasmic levels of Snail/Slug and number of neighboring cells, while increased cell area and eccentricity.

**Figure 8:**
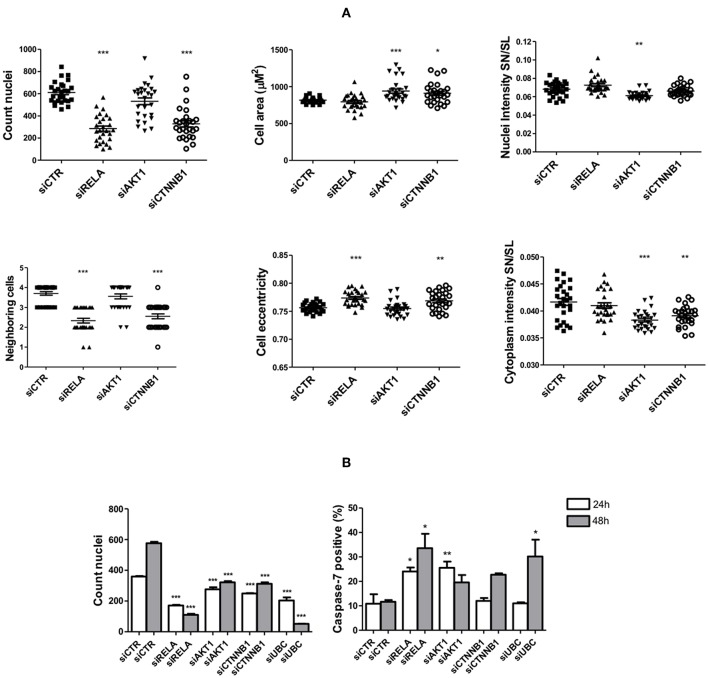
Changes in cell survival and EMT upon interferences in signaling pathways targeted by the anti-survival/EMT group. The FADU cell line was transfected with siRNAs specific for *RELA, AKT1, CTNNB1*, cytotoxic siUBC, and unspecific siRNA control (siCTR), followed or not by TNF-α stimulation. At the endpoint, images were acquired with an ImageXpress Micro XLS HCS system (Molecular Devices), using a 10X objective and excitation/emission filters DAPI (Hoechst), FITC (SN/SL) and Cy5 (CellMask Deep Red). **(A)** Cells were stimulated for 72 h with TNF-α (20 ng/mL), starting 24 h post-transfection, followed by immunostaining with antibodies against Snail/Slug (SN/SL) and co-staining with nuclear (Hoechst) and cytoplasmic (CellMask) dyes. Alterations in morphometric parameters, cell counts and nuclear/cytoplasmic Snail/Slug due to siRNA transfection as observed after image analysis. **(B)** 24 and 48 h after transfection, cells without TNF-α stimulation were immunostained with antibodies against cleaved Caspase-7 and co-stained with nuclear (Hoechst) and cytoplasmic (CellMask) dyes. Cell counts and percentage of apoptotic cells positive for active caspase-7 as observed after image analysis. Statistically significant differences (ONE-WAY Anova with Tukey post-test), in comparison to the control condition: ^*^p < 0.05; ^**^p < 0.01; ^***^p < 0.001.

Without TNF-α stimulation, the knockdown of the selected targets led to a reduction in cell counts as early as 24 h post-transfection, especially on cells transfected with siRELA, in which the impact was comparable to siUBC (a cytotoxic siRNA). At the same time point, the percentage of apoptotic cells (positive for cleaved caspase-7) transfected with siRELA and siAKT1 was around 25% higher than the control group (10%) but was unaltered on cells transfected with siCTNNB1, which also reduced the cell number. After 48 h of siRNA transfection, in comparison to 24 h after transfection, the number of cells in the control condition had almost doubled (indicating cell proliferation), while only slightly increasing in cells transfected with siAKT1 and siCTNNB1 and further decreasing in cells transfected with siRELA and siUBC. The percentage of apoptotic cells slightly decreased in cells transfected with siAKT1, while increased in cells transfected with siCTNNB1 (although not attaining significance), siRELA and siUBC (Figure 8).

## Discussion

Many of the functional studies conducted so far about the impact of small molecules in cancer cell survival disregards the presence of an inflammatory microenvironment, which is known to promote apoptosis resistance, epithelial to mesenchymal transition, among other phenotypic changes that promotes therapy resistance and disease recurrence (7).With that in mind, our study aimed to identify, through functional assays using an HCS approach, miRs and signaling pathways with the potential to suppress both cell survival and EMT features in HNSCC cells considering the presence of an inflammatory microenvironment. This approach should provide evidence if the effect of previously studied miRs translates or not to cancer cells under inflammatory stimuli, as well as to describe the effect of miRs with no known effect in HNSCC cells so far.

Initially, we demonstrated the capacity of TNF-α to promote a broad spectrum of phenotypic changes characterizing EMT, including an increase in nuclear expression of Snail/Slug, mesenchymal markers N-Cadherin and Vimentin, as well as cell eccentricity, inter-cell distancing and cell migration. Interestingly, our results of the western blot assay indicate that Snail, rather than Slug, might be involved in the induction of EMT driven by stimulation with TNF-α. Moreover, the increased levels of beta-catenin after stimulation with TNF-α also indicates a possible role, in our model, for the Wnt/beta-catenin pathway for the induction of EMT. Based on our observations, we concluded that the treatment of cells with TNF-α at 20 ng/mL is the best option to induce FADU cells to EMT, as it promoted strong changes in all parameters evaluated, as well as a superior induction to cell migration in comparison to a higher concentration.

Next, by performing an HCS-based miR screening, we investigated the capacity of 31 human miR mimics to alter phenotypic features related to cell survival and EMT in FADU cells induced to EMT by TNF-α stimulation. Overall, the results from this screening led us to identify four groups of miRs, namely G1a, G1b, G2, and G3, with distinct activity in promoting/inhibiting cell survival and EMT. Among them, two groups had characteristics of oncomiRs: G1a (pro-survival/EMT) and G3 (pro-survival) whereas the two remaining groups had characteristics of tumor suppressor miRs: G1b (anti-survival) and G2 (anti-survival/EMT). Noteworthy, G1b (anti-survival) also excerpted an pro-EMT effect, while G3 (pro-survival) excerpted a anti-EMT effect, however, as the effects of G1b and G3 in EMT could be a byproduct (i.e., secondary effect) of their alterations in cell survival, those groups were not classified regarding their alterations in EMT (32).

Among the miRs from G1b (anti-survival), it was previously found that miR-101 is downregulated in HNSCC tissues from different anatomical sites, besides having an anti-survival effect on HNSCC cell lines, including FADU (33–35). Additionally, studies conducted with esophageal squamous cell carcinoma (ESCC)-derived cell lines subjected to the ectopic expression of miR-22 (also from G1b) observed a reduction in cell survival and migratory/invasive potential (36, 37). On the other hand, the pro-survival group G3 was mainly composed by miRs from miR-302-367 cluster, which are traditionally associated with pluripotency and malignancy of germ cells tumors (38, 39). In the context of head and neck cancer, overexpression of miR-302a and miR-302b was found in cells derived from HNSCC with characteristics of cancer stem cells including self-renewal and the ability to generate heterogeneous cell populations (40).

The pro-survival/EMT G1a was composed of miRs that are traditionally involved in regulatory mechanisms linking inflammation and tumor progression, including elements of the miR-17-92 cluster, miR-21 (a HNSCC oncomiR) and miR-23a/24/27a cluster (41). Interestingly, Chang and coworkers also observed a pro-survival activity of miR-21, as well as increased expression of miR-21 in primary HNSCC compared to mucosal controls (42). Moreover, in the recent meta-analysis study performed by Lubov et al., it was observed that an increased expression of miR-21 is associated with poor outcome in HNSCC (14). Differently from G1a, G2 (anti-survival/EMT) was composed of miRs from miR-29 family, which displays tumor suppressor activity in several types of cancer, including HNSCC (14, 43). In line with our results, Kinoshita and coworkers observed that the ectopic expression of elements from the mir-29 family in the FADU cell line resulted in a significant reduction in cell number, as well as in cell migration and invasion (44).

In an effort to identify, among the several targets and signaling pathways regulated by miRs, those that effectively contributed to the observed phenotypic effect, we excluded from further analyses genes that were commonly targeted by groups of miRs that led to opposite phenotypes. Those filtered targets were used in *in silico* enrichment analysis, leading to the identification of specific targets and targeted signaling pathways. Noteworthy, this strategy provided cues on the genes and signaling pathways to be explored to suppress HNSCC tumor growth and metastasis.

By following this strategy, we observed that miRs from G1b (anti-survival) and G2 (anti-survival/EMT) targeted signaling pathways that are associated with the interface between inflammation and tumor initiation/progression, including MAPK, PI3K/AKT and NF-κB pathways (45). However, in comparison to predicted targets from G1b, it was found that miRs from G2 interfered in the PI3K/AKT and NF-κB pathways in a more extensive manner, also targeting a regulatory subunit of PI3K (PIK3R3), a oncogene that regulates AKT activity, as well as *RELA*, which codes for the canonical subunit (p65) of the NF-κB transcription factor (46, 47). Moreover, miRs from G2 also targeted several elements of the Wnt/beta-catenin signaling pathway. Altogether, our results from *in silico* analyses provided evidence that the anti-survival/EMT effects elicited by miRs from G2 likely derives from an extensive perturbation in PI3K/AKT and NF-κB pathways, besides Wnt signaling pathway. Additionally, by evaluating alterations in gene expression levels of cells transfected with miRs from G2 (anti-survival/EMT) group, we confirmed that elements from the following targeted signaling pathways: NF-κB (*RELA* and *IL6*), PI3K/AKT (*AKT2*), as well as Wnt/beta-catenin (*MYC*) were downregulated after the transfection in most cases.

Additional functional assays were performed with siRNAs against genes from the following signaling pathways regulated by anti-survival/EMT group (G2): *AKT1* (PI3K/AKT pathway); *RELA* (NF-κB pathway); and *CTNNB1* (Wnt pathway) followed or not by stimulation with TNF-α. Noteworthy, though *AKT1* and *CTNNB1* are not directly targeted by the miRs from G2, their use is justified by the central role of those genes in regulating the PI3K/AKT and Wnt/beta-catenin pathways, respectively, which were extensively targeted by those miRs. Therefore, results from our functional assays using siRNAs should not be interpreted as direct link between anti-survival/EMT miRs and a specific target, but rather between miRs and targeted signaling pathways. An exception are observations from siRELA transfections, as *RELA* is not only a central gene in the NF-κB pathway but also a direct target of G2.

By stimulating cells with TNF-α after siRNA transfection, we sought to investigate the individual role of NF-κB, PI3K/AKT, and Wnt/beta-catenin signaling pathways on either cell survival or EMT considering the presence of an inflammatory microenvironment. Interestingly, we found that although gene silencing of *RELA* and *CTNNB1* led to an anti-survival effect whereas silencing *AKT1* led to an anti-EMT effect, none of the siRNAs alone impaired both cell survival and EMT, which indicates that the effects of anti-survival/EMT miRs are most likely due to their potential to interfere in multiple signaling pathways simultaneously. This possibility points out to the potential benefits of a multi-target approach to treat HNSCC, especially considering that so far, clinical trials evaluating the capacity of PI3K inhibitors to treat HNSCC have shown disappointing results (48). In line, a recent study by Li et al. demonstrated that co-targeting EGFR (upstream of PI3K/AKT) and NF-κB pathways led to a superior inhibition of cell survival and xenograph tumor growth, when compared to targeting either pathway alone (49).

By not stimulating cells with TNF-α after siRNA transfection, we aimed to evaluate if the effects coming from the interferences in NF-κB, PI3K/AKT, and Wnt/beta-catenin signaling pathways are influenced by the presence of an inflammatory microenvironment. Additionally, we investigated if effects on cell survival are due to alterations in apoptosis by evaluating the percentage of cells positive for cleaved caspase-7. Strikingly, we found that transfection with siRELA not only dramatically reduced the number of cells but also strongly induced cell-death by apoptosis after 24 h and 48 h. This indicates that interferences in the NF-κB pathway is deleterious to HNSCC cells regardless of stimulation with inflammatory factors. Interestingly, transfection with siAKT1 also followed a similar pattern (although not further increasing apoptosis at 48 h) revealing that an inflammatory stimulation exerts a protective effect on HNSCC cells against the deleterious effect of interferences in the PI3K/AKT pathway. Moreover, although transfection with siCTNNB1 reduced cell count, it did not enhance the number of apoptotic cells 24 h post-transfection, indicating a more prominent role of Wnt signaling toward cell proliferation regardless of TNF-α stimulation.

As a whole, our study identified several molecules that may have the potential to be used for prognosis or miR-based targeted therapies against HNSCC considering the presence of an inflammatory microenvironment. By further investigating the miRs with anti-survival/EMT effects, we found that interferences in the signaling pathways: NF-κB and Wnt/beta-catenin were the ones that most likely contributed for the anti-survival effect, whereas interferences in PI3K/AKT signaling pathway was most likely associated with anti-EMT effect. Future studies using *in vivo* models should shed light into the anti-tumor and anti-metastatic activity of the miRs and targets herein identified.

## Conclusion

The present work characterized the functional role of a set of human miRs in modulating a broad spectrum of phenotypic alterations related to HNSCC cell survival and EMT in cells under an inflammatory stimulation, as well as the potentially involved signaling pathways. More specifically, the following miR mimetics: miR-24-3p, miR-29a-3p, miR-29b-3p, miR-302a-3p, miR-302a-5p, miR-372-3p, and miR-373-3p were identified as the ones with greatest potential use in microRNA replacement therapies, as they displayed an anti-survival/EMT effect. On the other hand, endogenous miRs herein identified as with pro-survival/EMT effects: miR-18a-5p, miR-18b-5p, miR-19a-3p, miR-19b-3p, miR-21-5p, miR-27a-3p, miR-30a-5p, were identified as the ones with greatest potential use in microRNA inhibition therapies. Additionally, we found that together, interferences on NF-κB, PI3K/AKT, and Wnt/beta-catenin signaling pathways are the ones that most likely driven the anti-survival/EMT effects displayed by miRs. Individual gene silencing of components of those pathways, namely *RELA* (NF-κB), *AKT1* (PI3K/AKT), and *CTNNB1* (Wnt/beta-catenin), partially recapitulated the effects displayed by miRs with anti-survival/EMT effects. Our findings revealed miRs and signaling pathways that might be explored to fight HNSCC tumor growth and metastasis considering the presence an inflammatory microenvironment.

## Author Contributions

BS, WA, VF, DC, MZ, and RP: study concept. BS, FS, and RP: study design. BS, FS, IM, JS, RP, AC, and CT: data acquisition, analysis, and interpretation. BS: statistical analysis. BS and RP: manuscript preparation and editing. BS, WA, VF, DC, MZ, and RP: manuscript review.

### Conflict of Interest

The authors declare that the research was conducted in the absence of any commercial or financial relationships that could be construed as a potential conflict of interest.
