# Supplementary material for: A High-Content Screening Approach to Identify MicroRNAs Against Head and Neck Cancer Cell Survival and EMT in an Inflammatory Microenvironment
Source: Front Oncol. 2019 Nov 8;9:1100. doi: 10.3389/fonc.2019.01100 (PMC6856008; doi:10.3389/fonc.2019.01100)
Supplement: Supplementary file 1 [file Data_Sheet_1.pdf]

Supplementary file 1: human miRs mimetics and siRNAs.

miR mimetic	Catalog number
hsa-miR-17-3p	PM12246
hsa-miR-18a-5p	PM12973
hsa-miR-18b-5p	PM10466
hsa-miR-19a-3p	PM10649
hsa-miR-19b-3p	PM10629
hsa-miR-20a-5p	PM10057
hsa-miR-20b-5p	PM10975
hsa-miR-21-5p	PM10206
hsa-miR-22-3p	PM10203
hsa-miR-23a-3p	PM10644
hsa-miR-24-3p	PM10737
hsa-miR-27a-3p	PM10939
hsa-miR-29a-3p	PM12499
hsa-miR-29b-3p	PM10103
hsa-miR-30a-5p	PM11062
hsa-miR-92a-3p	PM10916
hsa-miR-101-3p	PM11414
hsa-miR-106a-5p	PM12567
hsa-miR-145-5p	PM11480
hsa-miR-181d-5p	PM12522
hsa-miR-222-3p	PM11376
hsa-miR-302a-3p	PM10936
hsa-miR-302a-5p	PM12557
hsa-miR-302b-3p	PM10081
hsa-miR-302b-5p	PM12916
hsa-miR-302c-3p	PM10571
hsa-miR-302d-3p	PM10927
hsa-miR-363-3p	PM10149
hsa-miR-371a-3p	PM12262
hsa-miR-372-3p	PM10165
hsa-miR-373-3p	PM11024
Pre-miR miRNA Precursor Negative Control (PMC)	AM17110

siRNA	Catalog number
Silencer Select Negative Control No1 (siCTR)	4390843
siGENOME human UBC siRNA (siUBC)	#M-019408-01
Silencer Select AKT1	S660
Silencer Select CTNNB1	S438
Silencer Select GSK3B	S6241
Silencer Select RELA	S11914

Supplementary file 1: human miRs mimetics and siRNAs. Tables describing the human microRNA mimetics (Top) and siRNA molecules (bottom) utilized for this study.
