# Supplementary material for: A High-Content Screening Approach to Identify MicroRNAs Against Head and Neck Cancer Cell Survival and EMT in an Inflammatory Microenvironment
Source: Front Oncol. 2019 Nov 8;9:1100. doi: 10.3389/fonc.2019.01100 (PMC6856008; doi:10.3389/fonc.2019.01100)
Supplement: Supplementary file 2 [file Data_Sheet_2.pdf]

Supplementary file 2: Genes targeted by groups of miRs

G1a filtered targets	G2 filtered targets	G1b filtered targets	G3 filtered targets
AAED1	A1CF	ABRACL	A1CF
AAGAB	AADACL3	AC007375.1	ABCA1
ABCA1	AARD	AC012215.1	ABCA6
ABCB7	ABAT	AC135983.2	ABCB11
ABL1	ABCA10	ADAM22	ABCD2
ABLIM1	ABCA6	ADAM28	ABCF3
AC002472.13	ABCB11	ADAMTSL5	ABHD13
AC135178.1	ABCE1	AGAP1	ABHD15
ACACA	ABCF1	AGFG1	ABL2
ACP1	ABCF3	AIDA	ABR
ACSL3	ABCG2	AKT2	ABRA
ADAM11	ABCG8	AMER2	AC007952.5
ADAM22	ABHD15	AMOTL1	AC027763.2
ADAP2	ABHD5	ANK2	AC079210.1
ADARB1	ABL2	ANKMY1	AC106017.1
ADD2	AC005003.1	ANKRD52	AC137932.1
ADD3	AC006486.1	ANKRD9	ACADSB
ADNP	AC007390.5	ANKS6	ACBD7
ADRBK2	AC007952.5	APOBEC3F	ACER3
AEBP2	AC016559.1	AR	ACOT2
AGFG1	AC074091.13	ARAP2	ACSL6
AGPAT6	AC079602.1	ARHGAP17	ACTRT3
AKAP6	AC106017.1	ARL5A	ACVR2B
AKR7A2	AC127496.1	ATG7	ADAM19
AL353791.1	AC137932.1	ATP2A2	ADAMTS5
AL953854.2	ACADSB	ATP6V1E1	ADH1B
ANGPT2	ACBD7	ATP8A1	ADO
ANKIB1	ACOT9	B3GALT5	ADRBK2
ANO3	ACP6	B3GAT2	AFF1
ANXA5	ACPL2	BACE2	AFF2
AP3S2	ACSBG1	BEND4	AGAP9
APAF1	ACSM2A	BTRC	AGBL3
AR	ACSM2B	C10orf67	AGGF1
ARC	ACSS3	C12orf36	AGMAT
ARHGAP5	ACTN2	C15orf40	AGO2
ARHGEF12	ACTR1A	C17orf51	AGO3
ARID1A	ACTR8	C20orf112	AGO4
ARID4B	ACTRT3	C2orf15	AGPAT3

ASCL1	ACVR1C	C5AR2	AIFM1
ATF2	ACVR2B	C6orf120	AK3
ATG2B	ADAL	CACNA1D	AKAP5
ATOX1	ADAM19	CACNA1E	AKAP6
ATP6V1A	ADAM23	CADM1	AKR1D1
ATP8A2	ADAM33	CAMK2N1	AKR7A2
ATPAF1	ADAMTS13	CASD1	AL590235.1
ATXN7	ADAMTS4	CASP10	AL590483.1
ATXN7L1	ADCYAP1R1	CBL	ALDH6A1
AVPR1A	ADH1B	CCDC127	ALG14
B3GAT2	ADH5	CCDC85C	ALG9
B4GALT5	ADHFE1	CD302	ALKBH1
BBX	ADO	CD55	AMD1
BEND3	ADSL	CDC42EP3	ANAPC16
BMP2K	AGAP1	CDH2	ANGEL2
BMP3	AGFG2	CDK14	ANKRD12
BRD7	AGGF1	CDON	ANKRD13C
BTF3L4	AGO3	CDS2	ANKRD33B
BTG1	AGO4	CDV3	ANKRD50
BTN1A1	AGPS	CENPP	AP005482.1
C10orf105	AGTPBP1	CERCAM	AP1S3
C11orf45	AHCYL1	CHD7	AP3S2
C12orf66	AHNAK	CHRM3	AP4S1
C15orf38-AP3S2	AHR	CMBL	AP5M1
C16orf52	AHRR	CMSS1	APBB2
C17orf105	AHSA2	CMTM4	APOLD1
C17orf51	AICDA	CNTNAP5	APP
C17orf85	AIFM1	COL20A1	APTX
C1QTNF7	AK1	CPEB4	AQP4
C1orf21	AK4	CRTAP	AQR
C1orf210	AKAP2	CTC1	ARGFX
C2orf15	AKAP5	CTDSPL2	ARHGAP18
C2orf43	AKT2	CXorf56	ARHGAP29
C2orf48	AKT3	DCAF12	ARHGEF39
C2orf91	AL021546.6	DCP1A	ARHGEF5
C3orf17	AL590235.1	DCTN5	ARL1
C3orf70	AL590483.1	DDIT4	ARL10
C4orf33	AL590822.1	DDX6	ARPP19
C5orf51	AL590822.2	DGCR14	ARSG
C5orf63	ALDH5A1	DIEXF	AS3MT
C6orf62	ALX4	DNAH10OS	ASB4
CA13	AMER2	DSG3	ASPA
CAB39	ANGEL1	DTNA	ASTN1

CACNA1C	ANK2	E2F2	ASTN2
CALD1	ANKFY1	ECE1	ASXL2
CALN1	ANKRD13B	EIF2AK1	ASXL3
CAP1	ANKRD13C	EIF4E3	ATMIN
CASK	ANKRD17	ELAVL2	ATOX1
CBFB	ANKRD33B	ELAVL3	ATP11A
CBLN2	ANKRD45	ELFN2	ATP5A1
CCDC144A	ANKRD49	EMC10	ATP5G1
CCDC149	ANKRD52	EMCN	ATP5G3
CCL1	ANKRD62	EMP1	ATP5S
CCNT2	ANKS6	FAM13A	ATP8A2
CCPG1	ANO10	FAM211A	ATXN1
CD47	AP000695.1	FAM217B	AZIN1
CD55	AP005482.1	FAM73B	BAG2
CDC73	AP1S3	FBXO31	BCAP29
CDCP1	AP3M1	FEM1B	BCAT1
CDHR3	AP3M2	FMNL3	BCL10
CDK19	AP4S1	FOXO3	BCL2L11
CDS2	AP5B1	FOXP1	BCL2L2
CEACAM1	AP5S1	FRMD6	BCLAF1
CENPP	AP5Z1	G3BP1	BID
CEP120	APBB2	GABBR2	BMP8B
CEP57	APC	GABRB2	BNC2
CHD7	APCDD1	GABRG1	BPTF
CHRM3	APOL6	GATAD2B	BRMS1L
CHST15	APOOL	GATSL2	BRWD3
CLCN4	APPBP2	GDAP2	BTD
CLIC5	APTX	GINS2	BTG1
CLNK	AQP1	GLI3	BTLA
CLUAP1	AQP4	GNG12	C10orf118
CLVS2	AQP7	GOSR1	C12orf4
CMC1	AQR	GPI	C12orf5
CNPPD1	AREL1	GRB10	C12orf66
CNTNAP3	ARHGAP20	GRM1	C14orf142
CNTNAP3B	ARHGAP26	GSE1	C15orf38-AP3S2
CNTNAP5	ARHGAP29	GTF2B	C16orf72
COQ10B	ARHGAP31	GZF1	C18orf21
CORO2A	ARHGEF10	HCFC2	C18orf32
CPPED1	ARHGEF5	HCN4	C1orf50
CREBL2	ARL10	HNF4G	C21orf59
CREBRF	ARL11	HNRNPA3	C2CD2
CREG2	ARL13B	HS3ST1	C2orf69
CSMD1	ARL3	IKBKG	C3

CSRNP3	ARL4A	IKZF4	C4orf32
CTC-432M15.3	ARL4C	IPO9	C4orf40
CTGF	ARL5A	IST1	C7orf41
CTNNA3	ARL5B	ITGA3	C7orf60
CTSO	ARL6IP4	JPH3	C8A
CYP19A1	ARL8B	KATNBL1	C8orf44-SGK3
DDX18	ARMC8	KBTBD6	CADM2
DGKG	ARNT2	KCND3	CALCOCO2
DICER1	ARNTL2	KCNH1	CAMK4
DIP2A	ARPC4-TTLL3	KCNJ11	CAPRIN1
DIP2C	ARPP21	KCNK5	CAPZB
DIRAS2	ARRB1	KCNMB4	CASC4
DMD	ART4	KCNQ3	CBFA2T2
DNAAF2	AS3MT	KIAA0556	CBX5
DNAJC16	ASAP2	KIAA1456	CCDC141
DNAJC3	ASB13	KIAA1715	CCDC144A
DOCK4	ASB16	KIAA2022	CCDC170
DOCK5	ASB18	KIF3B	CCDC171
DST	ASCC1	KLHL30	CCDC25
EBF4	ASPA	KLHL4	CCDC58
EDIL3	ASTN1	KMT2A	CCDC6
EFR3A	ASXL3	KNSTRN	CCDC62
EGR3	ATAD2B	KPNA3	CCDC77
ELOVL4	ATCAY	KRT77	CCDC80
ELOVL5	ATG12	KSR2	CCND2
ELP2	ATG14	LIMCH1	CCNJ
EML5	ATIC	LMBR1	CCSER2
ENC1	ATOH8	LONP2	CD109
ENPP1	ATP11B	LRIG3	CD226
ENPP6	ATP1A2	LY75	CD28
ERCC6	ATP2B2	LY75-CD302	CD84
ERG	ATP2B4	MAPK10	CDC14B
ERLIN1	ATP5A1	MARVELD1	CDC73
ERVMER34-1	ATP5E	MASTL	CDKN2AIP
ESR1	ATP5J2-PTCD1	MCF2L	CECR2
ETNK1	ATP5S	MECP2	CELF1
ETV3	ATP6V0A2	MEGF11	CEP128
EVI5	ATP7B	METTL15	CEP135
EXOC6B	ATRN	MFAP3	CFD
EYA4	ATXN3	MGAT4A	CFL2
F3	AUNIP	MMP19	CHL1
FAIM3	AVL9	MPZL1	CHML
FAM104A	AVPI1	MRPL30	CHMP3

FAM110C	B3GNT5	MRPS23	CHRM2
FAM126B	B4GALT6	MRRF	CHST11
FAM136A	BACE2	MTHFD2	CIAO1
FAM160A1	BAG2	MTMR2	CLCN5
FAM160B1	BAHD1	MTMR3	CLDN19
FAM162B	BAMBI	MTUS2	CLEC2D
FAM167A	BBIP1	MUC19	CLEC4C
FAM168A	BCAS4	MXRA7	CLMN
FAM189A1	BCDIN3D	NANOS1	CLN8
FAM196A	BCL10	NAP1L1	CLNK
FAM199X	BCL11B	NCAPD3	CLPB
FAM213B	BCL2L11	NCMAP	CLSPN
FAM46B	BCL2L15	NDUFA5	CLSTN2
FAM73A	BCL2L2	NECAB3	CLTC
FAM8A1	BCL6B	NEU3	CLUAP1
FAXC	BCL7A	NFAT5	CNBP
FBXL3	BDKRB2	NFATC2	CNDP1
FBXO30	BEND4	NFIC	CNDP2
FBXO32	BICD1	NINJ1	CNNM2
FDX1	BID	NPNT	CNOT2
FIGN	BLOC1S3	NSD1	CNOT6
FKBP15	BLVRA	NT5DC1	CNTNAP2
FLT1	BMF	NUDT16	COLEC10
FNDC3A	BMP10	NUP93	COPS8
FOXP2	BMP7	NYAP2	COX20
FRAS1	BMPR1A	OBFC1	COX6B1
FXR1	BMPR1B	ONECUT1	COX7B
FZD8	BMS1	ONECUT2	CPE
G2E3	BNIP3L	P2RY2	CPM
GABRA4	BOD1L2	PAFAH1B2	CREB5
GAREM	BPHL	PAX7	CRIPT
GBP2	BPI	PEG10	CRISPLD1
GCC2	BPNT1	PEX2	CRISPLD2
GCNT2	BPTF	PGR	CSRNP3
GIPC3	BRCC3	PHEX	CTD-2368P22.1
GIT2	BRI3BP	PHF12	CTDSPL
GLCCI1	BRIP1	PIGP	CTH
GLS	BRMS1L	PIK3CD	CUL3
GNA12	BROX	PKIA	CYB5R4
GNAQ	BSND	PLA2G16	CYBRD1
GNPTG	BTBD7	PLA2G2C	CYP7B1
GOLGA6A	BTBD9	PPARGC1A	CYR61
GOLGA6B	BTLA	PPIL4	DBF4

GOLGA6C	BTN3A1	PPM1L	DBT
GOLGA6D	BYSL	PRDM16	DCAF17
GPAM	C10orf11	PRLR	DCC
GPBP1	C10orf118	PRR14L	DCP2
GPR157	C10orf54	PSMF1	DDHD1
GPR26	C11orf30	PTCD2	DDI2
GPR63	C11orf58	PTCD3	DENND5B
GREB1L	C12orf4	PTCHD4	DERL2
GRHL2	C12orf49	PTDSS1	DHFRL1
GRM1	C12orf5	PTPN3	DHTKD1
GRM4	C12orf52	PVRL1	DHX33
GRSF1	C14orf105	QSER1	DISC1
GSK3B	C14orf37	RAB30	DMGDH
GSPT1	C15orf38	RAP2B	DMP1
GSTM2	C15orf40	RAPGEF5	DMXL1
GTF2A1	C15orf57	RASGRF2	DNAJB4
GUCY1A3	C16orf72	RBM48	DNAJC10
GXYLT1	C17orf75	RBM8A	DNAJC18
HBS1L	C18orf21	RCL1	DNAJC21
HCFC2	C19orf40	RCN2	DNAJC27
HCN4	C1GALT1	RCSD1	DOCK11
HECTD3	C1ORF220	RGS6	DPM1
HELZ	C1QTNF6	RHBDL3	DPPA3
HERPUD2	C1orf168	RHCG	DPY19L4
HGF	C1orf220	RILPL1	DR1
HHIP	C1orf50	RMND5A	DRG1
HIPK3	C1orf52	RPL28	DSE
HMGCS1	C20orf112	RPL36A-HNRNPH2	DSTYK
HMGN2	C21orf49	RPS3	DUSP18
HMX1	C21orf58	RPS6KA5	DUSP6
HNRNPA0	C22orf29	RPTOR	DYRK2
HNRNPR	C22orf39	SARM1	EAF1
HOMER1	C2CD2	SC5D	EEF2K
HOXA9	C2CD4A	SEC22C	EFR3A
HRK	C2ORF15	SEC23IP	EHD3
HS3ST3A1	C2orf49	SEC63	EID2B
HS6ST1	C2orf68	SEMA6D	EIF3F
HSDL2	C2orf72	SERINC3	EIF3M
HSF5	C2orf88	SESN2	EIF4E
ICA1L	C3	SIGLEC9	EIF4EBP2
ID4	C4orf29	SKP1	EIF5
IGF1	C4orf3	SLAMF1	EIF5B
IGSF3	C4orf50	SLC14A1	ELK1

IL1RAP	C5orf20	SLC1A2	ELK4
IL1RAPL1	C5orf24	SLC22A23	ELOVL6
IL6ST	C5orf30	SLC24A1	EMC1
IL9R	C6orf120	SLC30A6	EMR2
INHBA	C6orf89	SLC35F6	ENAH
INSIG1	C7orf41	SLC36A1	ENPP5
INSIG2	C7orf60	SLK	ENSA
IPMK	C7orf65	SLMO2	ENTPD5
IREB2	C7orf73	SMC1A	EPC2
ITGA1	C8orf37	SNAP29	EPHA5
ITGA8	C8orf86	SNX24	EPS8
ITGB8	C9orf3	SNX30	EPT1
ITPA	C9orf47	SOCS2	ERBB4
IYD	CABP4	SORCS3	ERCC6L2
JAKMIP3	CACNA1I	SORL1	ERGIC2
JRKL	CACNG4	SOX6	ESCO2
KANSL3	CACNG8	SPCS2	ESR2
KATNAL1	CACUL1	SPECC1	ETNK1
KBTBD7	CADM1	SRSF10	EXO5
KCNJ15	CAMK2D	SRSF7	EXOC5
KCNK10	CAMK2N1	STC1	EXPH5
KCNS2	CAMKK2	STK38L	EXT1
KCNV1	CAMSAP1	STOX2	EYS
KCTD12	CAPN13	STRBP	FAM105B
KDM5B	CAPN7	STX4	FAM110C
KIAA0247	CAPZA2	STYX	FAM129A
KIAA1045	CARD18	SWAP70	FAM151B
KIAA1211	CASC4	SYAP1	FAM160B1
KIAA1462	CASD1	TADA2B	FAM169A
KIAA1614	CASP8	TAF8	FAM179A
KIF1B	CAV2	TBC1D24	FAM216B
KLF6	CBFA2T2	TBC1D30	FAM222B
KLF7	CCDC170	TET2	FAM46C
KLHL20	CCDC171	TET3	FAM47E
KLHL3	CCDC25	TGFBR1	FAM53B
KLHL4	CCDC3	THRB	FAM83F
KLHL42	CCDC6	THSD7A	FAM96A
KLHL5	CCDC69	TIMP3	FAM98B
KPNA3	CCDC80	TMEM178B	FAM9C
KRTAP4-9	CCDC88A	TMEM236	FANCA
LCLAT1	CCDC90B	TMEM65	FANCC
LEMD3	CCDC93	TNFAIP8	FARSB
LHX9	CCL28	TNFSF15	FAS

LIFR	CCNDBP1	TNKS2	FASLG
LIN28B	CCNJ	TP63	FAT3
LPGAT1	CCSER2	TPK1	FAXC
LRRTM2	CD109	TRAM2	FBXL20
MAGI2	CD300LB	TRAPPC9	FBXO21
MAN1A2	CD300LG	TRIM2	FBXO28
MAP3K1	CD58	TRIM27	FBXO30
MAPK8	CD63	TRIM35	FBXO48
MARCH5	CD83	TROVE2	FECH
MARCH6	CD93	TTYH3	FEM1A
MASTL	CD99L2	TXLNA	FGD4
MBD2	CDC23	UBE2F	FGD6
MBNL1	CDC42BPA	UBE2N	FGF2
MBNL2	CDC42EP3	UNC5D	FGL2
MC2R	CDC42EP4	UQCC1	FHL2
MCHR2	CDC5L	USP46	FKBP14
MDGA1	CDH1	VPS13D	FKTN
MED13	CDH6	WDR41	FLVCR1
MED9	CDK12	WEE1	FMN1
MEF2D	CDK2	WNT2B	FNBP1L
MEI4	CDK9	WT1	FNDC3B
MEIS1	CDKN2AIP	WWTR1	FNIP2
MESDC2	CDKN2AIPNL	XPR1	FOSL2
MFAP3L	CDKN2B	XRCC3	FOXB1
MFSD6	CDX2	XYLT1	FOXK1
MID1IP1	CECR1	YAP1	FRRS1L
MLK4	CELF2	ZBED4	FRS2
MMGT1	CELF3	ZC3H6	FSD1L
MMP16	CENPBD1	ZDHHC14	FUBP1
MR1	CEP104	ZFP28	FUCA2
MRPL30	CEP72	ZFP62	FUNDC2
MRPL35	CEP76	ZHX3	FZD3
MSH2	CEP78	ZNF106	FZD4
MSI2	CEP85L	ZNF19	G6PC
MSRB2	CEP97	ZNF317	GABPB2
MTR	CERK	ZNF407	GABRA2
MUT	CERS4	ZNF576	GABRB1
MXRA7	CFD	ZNF705G	GATA6
NACC2	CFHR4	ZNF736	GCLM
NAV1	CHCHD5	ZNF749	GCNT4
NCKIPSD	CHD5	ZNRF3	GDE1
NEDD4	CHM		GEMIN4
NEO1	CHST11		GEN1

NEU3	CHST14		GFPT1
NEURL1B	CHURC1		GGCX
NEUROD1	CINP		GINM1
NFASC	CISD2		GJA3
NFAT5	CKAP2L		GK5
NFIC	CKS1B		GLIPR1
NHS	CLCN6		GLRA3
NHSL2	CLDN1		GLYAT
NIPAL2	CLEC16A		GM2A
NKAIN1	CLEC4C		GMEB1
NPL	CLMN		GNL3L
NRG1	CLN8		GNS
NUDT13	CLPX		GOLGA1
NUDT21	CLSPN		GOLGA8A
NUP62CL	CLSTN2		GOLGA8B
NXT2	CLTC		GOLGA8H
OCRL	CMBL		GOLGA8I
OGFOD3	CMKLR1		GOLGA8J
OLFM3	CMPK1		GOLGA8K
OLR1	CNBP		GOLGA8M
ONECUT2	CNDP2		GOLGA8N
ORC2	CNIH1		GOLGA8O
OTUD7A	CNNM2		GOLGA8R
P2RY2	CNNM3		GPBP1
PAPD5	CNR2		GPC6
PARP15	CNTLN		GPR137B
PATE2	CNTNAP2		GPR137C
PATL1	COA1		GPR155
PAX6	COG5		GPR158
PCTP	COL25A1		GPR161
PCYT1A	COL4A2		GPR180
PDE11A	COL4A4		GPR37
PDE4DIP	COL5A1		GPR75
PDE5A	COL6A6		GPRIN2
PDGFC	COL9A1		GPRIN3
PDGFRB	COLEC10		GPX8
PDK1	COMMD2		GRAP2
PDPK1	COMMD9		GRIN2B
PDPR	COPS3		GSTCD
PEX5L	COQ7		GTF2H5
PHF12	CORO1C		GTPBP10
PHF13	COX10		GUCY1A2
PHF19	COX20		GUCY1A3

PHIP	COX7B		GXYLT1
PIK3C2A	CPM		GXYLT2
PITPNM2	CR1		H2AFV
PLAA	CRAMP1L		HARBI1
PLCL1	CREB3L2		HAS2
PLEKHA8	CREB5		HCN1
PLEKHM3	CREBBP		HDX
PLSCR1	CRIPT		HECW1
PNRC1	CRISPLD1		HECW2
POLR3F	CRISPLD2		HERPUD2
POT1	CRX		HES2
PPFIA2	CSF2RA		HEYL
PPIL4	CSNK1A1		HFE
PPIP5K2	SNK2B-LY6G5B-1181		HIST1H2BG
PPP1R3B	CTD-2545M3.6		HIST1H2BJ
PQLC2	CTDSPL		HLF
PREX2	CTH		HMBOX1
PRKAA2	CTNNBL1		HMGA2
PRKACB	CTRC		HMGB1
PRLR	CTSS		HMGB2
PRMT7	CTTNBP2NL		HNF4A
PSD3	CUL3		HNRNPA0
PSMF1	CUX2		HNRNPU
PTBP3	CXXC4		HOOK3
PTCH1	CXorf56		HS2ST1
PTCHD1	CYB561D1		HSD11B1
PTGER2	CYB5B		HSDL2
PTGFRN	CYB5R3		HSPA2
PTPN14	CYBRD1		HSPE1-MOB4
PTPRT	CYCS		IDS
PTRH2	CYP20A1		IFNE
RAB11FIP2	CYP27C1		IFRG15
RAB1A	CYP2B6		IKZF2
RAB4A	CYP2U1		IKZF3
RABGAP1	CYP4F3		IL17RA
RAD51	CYP4F31P		IL1R1
RALGAPA2	CYP4V2		INADL
RAP1A	CYTH3		INIP
RAP2B	CYTH4		INO80
RAPGEF5	DAAM2		INPP4A
RAPGEF6	DAK		INSIG2
RASA2	DAP3		INSR
RASEF	DBF4		INTU

RCOR1	DCAF12		IPCEF1
REEP1	DCAF16		IPP
RFT1	DCAF17		IRF2BP2
RGMB	DCAF5		IRGQ
RGPD4	DCDC2		ITPRIPL2
RGPD5	DCLRE1B		IYD
RGPD6	DCUN1D1		JAZF1
RGPD8	DCX		JMY
RGS7BP	DDA1		KAT2B
RIC3	DDHD1		KBTBD12
RIMS2	DDX4		KBTBD8
RLIM	DDX6		KCMF1
RNASEH2B	DENND1A		KCNB1
RNF111	DENND5B		KCNH5
RNF146	DENND6A		KCNJ6
RNF19B	DENR		KCNK6
RNF217	DFFA		KCNMB1
RNF44	DGKB		KDM1B
ROBO2	DGKI		KDSR
ROR1	DGKQ		KIAA0101
RP1-170O19.20	DHTKD1		KIAA0319
RP11-204N11.1	DHX30		KIAA0586
RPL12	DHX36		KIAA0930
RPS19	DIAPH2		KIAA1143
RPS3	DIP2B		KIAA1244
RPS6KA5	DISC1		KIAA1468
RSF1	DIXDC1		KIAA1551
RTKN2	DKK3		KIAA1586
RUFY3	DLD		KIAA1671
RUNX3	DLG3		KIAA1919
RXRA	DMBX1		KIAA1958
SAMD12	DMGDH		KIAA2018
SAMD8	DNAH17		KLF17
SAP30L	DNAJA2		KLF2
SAR1A	DNAJB14		KLF3
SCAI	DNAJB4		KLF6
SCAMP1	DNAJC24		KLF8
SCN9A	DNAJC28		KLHDC10
SDHAF2	DNAL1		KLHL15
SDK2	DNMT3A		KLHL23
SDPR	DOCK7		KLHL24
SEC61A1	DOK6		KLHL28
SEC63	DPM1		KLHL3

SECISBP2L	DPP8		KLHL31
SEL1L	DPY19L4		KLHL5
SEL1L3	DPYSL2		KLRC4
SEMA3A	DPYSL5		KPNA4
SEMA6D	DRAM1		KPNA5
SEPT7	DRAXIN		KY
SERPINB5	DRD1		LAMP2
SESN3	DSE		LAMTOR3
SESTD1	DSG2		LAX1
SFT2D3	DSG3		LCOR
SGCD	DSN1		LDLRAD4
SGIP1	DSTYK		LGI2
SGMS2	DTD2		LGSN
SH3KBP1	DTWD2		LHFPL2
SH3RF1	DTX4		LIMD1
SH3RF2	DUSP18		LIN28A
SHC3	DUSP2		LIN52
SHISA6	DUSP22		LIN54
SHROOM3	DVL3		LIPG
SIGLEC14	DYM		LLPH
SIK3	DYRK2		LMO3
SIPA1L1	DYRK3		LMX1B
SKA2	DZANK1		LOH12CR1
SKP1	E2F1		LONRF3
SKP2	E2F7		LPHN3
SLC12A6	EFNA5		LPIN1
SLC1A2	EFNB3		LPP
SLC24A4	EFTUD2		LPPR5
SLC25A46	EHD1		LRCH1
SLC35A5	EID1		LRP8
SLC35E2	EIF2AK2		LRPAP1
SLC6A20	EIF2S1		LRRC2
SLC6A6	EIF3F		LRRC27
SLC9A2	EIF3M		LRRC40
SLX4IP	EIF4E3		LRRC58
SMAD4	EIF5		LRRC8B
SMG1	ELF2		LSAMP
SNAP25	ELK1		LUZP2
SND1	ELMO2		LYPLA1
SNTG1	ELOVL6		LYRM7
SNX29	EMC3		LZIC
SOCS6	EMC7		MACC1
SOX12	EMR2		MAGEB10

SOX21	ENAM		MAGI3	
SPEN	ENPEP		MAP2K6	
SPICE1	ENSA		MAP3K2	
SPOCK1	ENTPD1		MAP3K9	
SRGAP1	ENTPD5		MAP4K2	
SRGAP3	ENTPD7		MAPK1	
SRPX2	EP400		MAPK14	
ST6GAL1	EPM2AIP1		MAPK1IP1L	
ST8SIA4	EPS8		MARCH5	
STAM2	EPT1		MARK1	
STAT2	ERBB2IP		MARVELD3	
STEAP2	ERBB3		MBD2	
STK39	ERCC4		MBNL3	
STOX2	ERCC6L		MBOAT2	
SULT1B1	ERGIC2		MCC	
SV2B	ERLIN2		MCOLN2	
SYNCRIP	ERN1		MCTP2	
SYNM	ERO1LB		MCTS1	
SYPL1	ESF1		MDFIC	
SYT15	ESR2		MDM2	
TAB3	ETV1		MDM4	
TBC1D1	EVI2B		MED12L	
TBL1X	EXOC3		MED28	
TBPL1	EXOC5		MEF2C	
TENM3	EXOC8		METAP1	
TENM4	EXOG		METTL20	
TGFBR3	EXOSC2		METTL24	
THBD	EXPH5		METTL2B	
THRB	EXT1		MFAP3L	
TIMM50	EYS		MFSD8	
TLR5	F2R		MGLL	
TLR8	FADS6		MIER3	
TM9SF3	FAM101A		MKL2	
TMEM110	FAM102B		MLTK	
TMEM164	FAM105B		MOB4	
TMEM237	FAM109B		MON2	
TMEM25	FAM114A1		MORC3	
TMEM254	FAM118A		MPLKIP	
TMEM39A	FAM120AOS		MR1	
TMEM64	FAM120C		MRE11A	
TMEM98	FAM131B		MRPL17	
TMX1	FAM13B		MRPL3	
TMX4	FAM151B		MRPS16	

TNFAIP3	FAM169A		MRPS25
TNFRSF10D	FAM177B		MRPS30
TNFSF14	FAM186B		MSANTD3
TNKS	FAM210B		MSANTD4
TNRC6A	FAM212B		MSRB3
TOMM20	FAM217B		MT1A
TOR1AIP2	FAM218A		MTA3
TOX4	FAM227A		MTDH
TP63	FAM229B		MTF1
TPD52	FAM3C		MTMR10
TRIM13	FAM63B		MTMR9
TRIM2	FAM71F2		MTO1
TRPA1	FAM78A		MYLK
TSHZ3	FAM83D		MYNN
TTC5	FAM84B		MYO18A
TTF2	FAM89A		MYO9A
TTPAL	FAM96A		MYOCD
TWISTNB	FAM98B		MYPN
TXLNA	FANCC		MYSM1
TXLNG	FANCM		NAA38
TXNDC12	FAS		NAALAD2
TXNRD3NB	FASTKD2		NABP1
UBE2F	FBLIM1		NBEAL1
UBE2K	FBXL18		NCBP1
UBE4A	FBXL2		NCKAP1
UBXN10	FBXL20		NCOA3
UNC80	FBXL4		NCOA7
USP37	FBXO40		NDUFAF5
USP6	FBXO41		NEK10
VAT1L	FBXO45		NFYB
VCPIP1	FBXO48		NIN
VGLL3	FBXO9		NINL
VKORC1L1	FECH		NIPAL1
VPS53	FER		NKAP
VSIG10	FGF2		NOM1
VTI1B	FGF9		NOS1AP
VWA1	FGFR1		NPFFR1
WAC	FGFR1OP		NR2F6
WBP4	FGL2		NR4A3
WDFY2	FHDC1		NUAK1
WDR33	FHL5		NUDT3
WHSC1L1	FITM2		NUP155
WNK3	FKBP14		NUP43

XCR1	FKTN		NWD1
XYLT1	FLJ27365		NXPE1
ZBTB10	FMNL3		NXPE3
ZBTB39	FOS		OCIAD2
ZC3H12B	FOXJ2		ONECUT3
ZCCHC3	FOXJ3		OTUD3
ZDHHC2	FOXK1		OTUD4
ZDHHC23	FOXK2		OXTR
ZFAND5	FOXO3		P2RX7
ZFP3	FOXP1		P2RY1
ZFP37	FRMD4B		P4HA2
ZFP62	FRRS1		PAFAH1B1
ZG16	FRRS1L		PAG1
ZNF10	FSBP		PALLD
ZNF100	FSTL1		PANK1
ZNF217	FUNDC2		PANX1
ZNF280B	FUT11		PAOX
ZNF35	FUT9		PAPD5
ZNF354C	FXN		PAPOLA
ZNF367	FZD4		PAPOLB
ZNF423	FZD5		PARK2
ZNF45	G3BP1		PARP2
ZNF451	G6PC		PARVA
ZNF514	GAB2		PAWR
ZNF566	GAL3ST3		PCDH1
ZNF609	GALC		PCDH10
ZNF618	GALNT10		PCNXL2
ZNF619	GALNT15		PDCD4
ZNF681	GALNT4		PDE12
ZNF703	GALNT6		PDE3B
ZNF766	GATAD1		PDE7B
ZNF778	GATC		PDHX
ZNF784	GATSL2		PDP2
ZNF788	GBP4		PEAK1
ZNF805	GBX2		PEX26
ZNF90	GCNT4		PEX5L
ZNRF2	GDAP1		PFKFB2
ZZZ3	GDF11		PGM2L1
	GDF5OS		PHACTR4
	GEMIN4		PHAX
	GFM1		PHF8
	GFPT1		PHIP
	GFRA1		PHLPP2

	GGA2		PI15
	GGCX		PIK3AP1
	GID8		PIK3C3
	GINS2		PIK3R3
	GINS4		PIKFYVE
	GIPC2		PKNOX1
	GJC1		PLA2G12A
	GK5		PLAG1
	GLB1L		PLD5
	GLDN		PLEKHM3
	GLIS3		PLRG1
	GLRA3		PLXDC2
	GLRX2		PMVK
	GLUL		PNPLA3
	GM2A		POC1B-GALNT4
	GMEB1		POFUT2
	GMFB		POLM
	GNAL		POLR3F
	GNG11		POLR3G
	GNG12		POU2F1
	GNL3L		POU6F2
	GNS		PPAP2B
	GOLGA3		PPARA
	GOLGA7		PPARGC1B
	GOPC		PPIC
	GOSR1		PPM1K
	GPATCH11		PPP4R1L
	GPCPD1		PPP4R2
	GPN2		PPP6C
	GPR155		PRKAA2
	GPR158		PRKAB2
	GPR161		PRKCB
	GPR61		PRKX
	GPR75		PRND
	GPRC5B		PRPF4
	GRAMD2		PRR13
	GRAP2		PRRG4
	GREM2		PSD3
	GRIA1		PSD4
	GRIK3		PTAFR
	GRIN3A		PTAR1
	GRM6		PTBP2
	GSR		PTEN

	GSTM3		PTGIS
	GTDC1		PTGR2
	GTF2H3		PTK2
	GTPBP10		PTPN14
	GUCY1A2		PTPN4
	GUF1		PTPRT
	GXYLT2		PURA
	H2AFX		PVR
	HARBI1		PYGO1
	HAS2		QKI
	HAS3		RAB21
	HAUS3		RAB23
	HAVCR1		RAB3B
	HBP1		RAB4A
	HCN1		RAB8B
	HDAC4		RABL5
	HEATR5A		RAD23B
	HECW1		RAD51L3-RFFL
	HELLS		RAD9B
	HEMK1		RAPGEF2
	HEYL		RASAL2
	HFE		RASSF2
	HHIPL1		RASSF6
	HIF3A		RBAK
	HINT3		RBFOX2
	HIP1		RBM25
	HIST1H2BG		RBMS3
	HIST1H2BN		RCAN3
	HLA-DOA		RDH13
	HLA-DOB		RDX
	HM13		REL
	HMGB1		REST
	HMGB2		RFFL
	HN1		RGL1
	HNF4G		RGP1
	HNRNPL		RGS17
	HNRNPUL1		RGS18
	HOXD4		RIF1
	HPSE		RIT1
	HRH1		RNF103-CHMP3
	HS3ST1		RNF11
	HS3ST3B1		RNF141
	HSD11B1		RNF149

	HSD17B12		RNF152
	HSPA1B		RNF180
	HSPA2		RNF19B
	HSPA5		RNF38
	HSPE1-MOB4		RNF8
	HTR2A		RORA
	HUNK		RORB
	IBA57		RP11-156E8.1
	ICMT		RP11-422N16.3
	ICOSLG		RPAP2
	IDE		RPL24
	IDS		RPL37
	IER5		RPP14
	IFFO1		RPS6KA3
	IFNAR1		RRAGD
	IFNAR2		RRN3
	IGF1R		RRP15
	IGF2BP1		RRP36
	IGHMBP2		RSAD2
	IKBKG		RSBN1
	IKZF1		RSF1
	IKZF3		RSPH3
	IL17RA		RXFP1
	IL17RB		RYK
	IL1R1		S1PR1
	IL1RL1		SAMD12
	IL21R		SAMD8
	IL6R		SAP30
	IL7R		SAR1B
	ILDR1		SASH1
	ILF3		SATB2
	IMP4		SBNO1
	IMPA1		SCIN
	IMPA2		SCN3B
	IMPG2		SCN8A
	INIP		SCO1
	INPP4B		SCRG1
	INTU		SDC2
	IP6K1		SDHAF1
	IPCEF1		SEL1L
	IPO8		SELPLG
	IPO9		SELT
	IPPK		SEMA3A

	IRF2BP2		SEMA3E
	IRGQ		SEMA4F
	IRS4		SENP1
	IRX4		SENP5
	ISCA2		SERINC1
	ISG20L2		SERPING1
	ISPD		SERTAD2
	ISY1		SESN3
	ITGA9		SFPQ
	ITIH5		SFT2D3
	ITPK1		SFXN1
	ITPKB		SGK3
	ITPRIP		SH3BP2
	ITPRIPL2		SH3BP5
	JAM2		SH3GLB1
	JAZF1		SH3TC2
	JDP2		SHE
	JMJD1C		SIGLEC6
	JMY		SIK1
	JOSD1		SIK2
	JPH2		SIKE1
	KARS		SIRPA
	KAT6A		SLC10A7
	KATNBL1		SLC15A2
	KBTBD12		SLC16A10
	KBTBD8		SLC16A12
	KCMF1		SLC16A6
	KCNA1		SLC1A5
	KCNB1		SLC22A15
	KCNC2		SLC24A2
	KCNC3		SLC25A16
	KCNJ2		SLC25A32
	KCNK1		SLC25A36
	KCNK5		SLC25A51
	KCNK6		SLC28A1
	KCNMB1		SLC2A3
	KCTD20		SLC30A1
	KDELC2		SLC30A10
	KDELR1		SLC30A7
	KDM5A		SLC33A1
	KDSR		SLC35A3
	KIAA0141		SLC35E1
	KIAA0226		SLC38A1

	KIAA0355		SLC38A7
	KIAA0391		SLC45A4
	KIAA0930		SLC4A4
	KIAA1143		SLC4A5
	KIAA1191		SLC5A3
	KIAA1377		SLC5A7
	KIAA1456		SLC7A11
	KIAA1468		SLC7A2
	KIAA1549L		SLC7A6
	KIAA1551		SLC8A3
	KIAA1586		SLC9A2
	KIAA1644		SLC9A7
	KIAA1671		SLFN12
	KIAA1919		SLFN12L
	KIAA1958		SLFN5
	KIAA2018		SLMAP
	KIF11		SMAD9
	KIF14		SMIM12
	KIF1A		SMU1
	KIF26B		SNAP47
	KIF2A		SNRPD1
	KIF3B		SNRPD3
	KIF5A		SNTB2
	KIF5B		SNX2
	KIF6		SOBP
	KIN		SOCS5
	KIRREL		SORT1
	KLB		SOX4
	KLF8		SP3
	KLHDC7A		SPATA13
	KLHDC8A		SPHAR
	KLHL18		SPOCK1
	KLHL23		SPOPL
	KLHL24		SPPL2A
	KLHL8		SPRY1
	KLK3		SPRY4
	KLLN		SREK1IP1
	KNSTRN		SRGAP1
	KPNA1		SRGAP2
	KRBA2		SRI
	KREMEN1		SRSF1
	KY		SRSF3
	LAIR1		SSBP2

	LAMC1		SSR1
	LAMC3		SSTR2
	LAMTOR3		ST6GAL2
	LANCL1		ST6GALNAC3
	LBP		ST8SIA3
	LCOR		STARD4
	LDHA		STON2
	LDHD		STRIP2
	LDLRAD3		STRN
	LDLRAD4		STRN3
	LDOC1L		STX17
	LEFTY2		STX3
	LEP		STX7
	LGI2		SUV39H2
	LGSN		SUV420H1
	LHFPL2		SYNPO2
	LHX6		SYNRG
	LIG3		TAL2
	LIMS1		TAOK1
	LIN28A		TBC1D8B
	LIN52		TBCEL
	LINC00908		TCEB1
	LIPG		TCEB3
	LIPT2		TERF2
	LL22NC03-63E9.3		TFDP2
	LMAN2L		TFEC
	LMLN		TGFBR2
	LMO4		TGIF1
	LMX1A		THAP1
	LOH12CR1		THUMPD3
	LONP2		TIAL1
	LPAR3		TIGD2
	LPCAT2		TIMD4
	LPIN1		TIMM50
	LPPR4		TIRAP
	LRAT		TLR4
	LRCH3		TMCC1
	LRG1		TMED5
	LRIG2		TMED7
	LRP6		TMEM106B
	LRRC2		TMEM120B
	LRRC27		TMEM135
	LRRC28		TMEM170A

	LRRC58		TMEM170B
	LRRD1		TMEM185B
	LSG1		TMEM194B
	LSM11		TMEM200C
	LSM14B		TMEM251
	LSM3		TMEM26
	LUC7L3		TMEM33
	LUZP2		TMEM56
	LY6G5B		TMEM59
	LYN		TMF1
	LYPLA1		TMOD2
	LYRM2		TMOD3
	LYRM7		TMPRSS11B
	LZIC		TMTC1
	MACC1		TNFAIP8L3
	MAGI1		TNFRSF10B
	MAGT1		TNR
	MAMDC4		TPGS2
	MANEA		TRABD2B
	MANEAL		TRAF1
	MAP1B		TRAF3IP2
	MAP1LC3B		TRAF6
	MAP3K15		TRDMT1
	MAP3K9		TRHDE
	MAP6D1		TRIM24
	MAP7		TRIM33
	MAPK10		TRIM36
	MAPK14		TRIM71
	MAPK1IP1L		TRIM72
	MAPK9		TRIP11
	MAPKAPK5		TRPM3
	MAPKBP1		TRPM7
	MAPRE2		TSC22D2
	MARCH7		TSKU
	MARK4		TSPAN6
	MAU2		TSPYL1
	MBL2		TTC28
	MBLAC2		TTC38
	MBNL3		TTC39B
	MCC		TTC9
	MCCC2		TTLL7
	MCL1		TTPAL
	MCM9		TVP23C

	MCMDC2		TWF1
	MDM2		TXK
	MDM4		TXNDC15
	MECR		TYRP1
	MED16		TYW5
	MED17		UACA
	MED28		UBC
	MED31		UBE2D3
	MED7		UBE2J1
	MED8		UBE2Q2
	MEF2A		UBIAD1
	MEGF8		UBXN2A
	MELK		UCHL3
	METTL14		UHMK1
	MEX3A		UHRF1BP1
	MEX3B		ULK1
	MFAP2		UQCR11
	MFN1		UQCRFS1
	MFSD2A		USP13
	MFSD8		UST
	MGAT1		UVRAG
	MGAT4A		UVSSA
	MGAT5		VAMP4
	MGLL		VASH2
	MIB1		VEZT
	MICA		VGLL2
	MICB		VHLL
	MIEF2		VIL1
	MIER3		VKORC1L1
	MKI67IP		VLDLR
	MKL2		VMA21
	MLLT10		VN1R1
	MLXIP		VPS4B
	MMADHC		VPS53
	MMP24		VSTM4
	MOB4		VWA2
	MOBP		VWC2
	MOCOS		WDFY2
	MOG		WDR36
	MPV17L		WDR72
	MRE11A		WHSC1
	MRGBP		WHSC1L1
	MRI1		WRN

	MROH6		WSB1
	MRP63		XIAP
	MRPL3		XKR4
	MRPL42		XPNPEP3
	MRPL48		XRCC5
	MRPL51		XRN1
	MRPS10		XRRA1
	MRPS16		YIPF4
	MRPS23		YPEL1
	MRRF		YTHDC1
	MRTO4		YTHDF3
	MS4A7		YWHAZ
	MSL1		ZADH2
	MSRB1		ZBTB18
	MTAP		ZBTB20
	MTCH2		ZBTB25
	MTDH		ZBTB33
	MTG2		ZBTB44
	MTMR10		ZBTB8A
	MTMR4		ZBTB8B
	MTMR9		ZC2HC1C
	MTO1		ZC3H12C
	MVB12B		ZCCHC8
	MXD1		ZDHHC15
	MXI1		ZDHHC3
	MYCN		ZFAND5
	MYH9		ZFP14
	MYLK		ZFP36L2
	MYO18A		ZFP91
	MYO6		ZFPM2
	N4BP1		ZFYVE9
	N4BP2		ZKSCAN8
	N4BP2L2		ZMYM1
	NAA25		ZMYM2
	NAA40		ZMYM4
	NAALAD2		ZNF107
	NABP1		ZNF117
	NANOS1		ZNF132
	NAP1L6		ZNF141
	NARF		ZNF148
	NBEAL1		ZNF207
	NCAPG2		ZNF223
	NCBP1		ZNF225

	NCMAP		ZNF234
	NCOA3		ZNF236
	NCOA7		ZNF250
	NDN		ZNF264
	NDST1		ZNF274
	NDUFA10		ZNF277
	NDUFAF5		ZNF28
	NDUFC2		ZNF284
	NDUFV3		ZNF285
	NECAP2		ZNF321P
	NEGR1		ZNF326
	NEK10		ZNF329
	NEK3		ZNF33A
	NEK8		ZNF35
	NETO2		ZNF354B
	NEUROG3		ZNF37A
	NF2		ZNF398
	NFATC2		ZNF417
	NFATC3		ZNF426
	NFATC4		ZNF43
	NFE2L1		ZNF430
	NFRKB		ZNF431
	NFYA		ZNF436
	NGFRAP1		ZNF461
	NHLRC2		ZNF468
	NICN1		ZNF470
	NINL		ZNF473
	NIP7		ZNF485
	NIPA2		ZNF486
	NIPAL1		ZNF514
	NKIRAS2		ZNF519
	NKX6-3		ZNF525
	NLN		ZNF546
	NLRP9		ZNF554
	NMNAT1		ZNF555
	NMT2		ZNF557
	NOA1		ZNF558
	NOL12		ZNF559
	NOL9		ZNF566
	NOM1		ZNF578
	NOS1		ZNF585A
	NOS1AP		ZNF587
	NPR1		ZNF589

	NPY4R		ZNF594	
	NQO2		ZNF597	
	NR2C2		ZNF607	
	NR3C1		ZNF611	
	NT5C2		ZNF621	
	NTMT1		ZNF639	
	NTRK2		ZNF641	
	NTSR1		ZNF660	
	NUDT11		ZNF667	
	NUDT16		ZNF669	
	NUP205		ZNF677	
	NUP43		ZNF682	
	NUP93		ZNF699	
	NUPL2		ZNF704	
	NWD1		ZNF708	
	NXPE3		ZNF716	
	OCIAD2		ZNF726	
	ODF2		ZNF766	
	OIP5		ZNF791	
	OLFM2		ZNF8	
	OLFML2A		ZNF805	
	ONECUT3		ZNF813	
	OPHN1		ZNF816	
	OR7D2		ZNF831	
	ORAI2		ZNF84	
	ORC6		ZNF85	
	ORMDL3		ZNF850	
	OSBPL6		ZNF860	
	OSBPL8		ZNF878	
	OTOG		ZRANB1	
	OTUD1		ZRANB3	
	OTUD4		ZRSR1	
	OXA1L			
	OXR1			
	OXTR			
	P2RX7			
	P2RY1			
	P2RY8			
	P4HA2			
	PABPC4L			
	PACRGL			
	PAICS			
	PAIP2B			

	PAK7			
	PALM2			
	PALM2-AKAP2			
	PAN2			
	PANK1			
	PANK3			
	PAOX			
	PAPOLA			
	PAQR5			
	PAQR6			
	PARD6G			
	PARG			
	PARP2			
	PARP8			
	PARVA			
	PAWR			
	PAX1			
	PBOV1			
	PBRM1			
	PCDH15			
	PCDH17			
	PCDHB2			
	PCDHB5			
	PCF11			
	PCGF5			
	PCP4L1			
	PCYOX1			
	PCYT1B			
	PDE3A			
	PDE3B			
	PDE6A			
	PDE7A			
	PDE7B			
	PDF			
	PDHB			
	PDHX			
	PDIK1L			
	PDLIM3			
	PDP2			
	PELP1			
	PER1			
	PEX13			
	PGBD1			

	PGLS			
	PGM2L1			
	PGM3			
	PGPEP1			
	PHACTR2			
	PHACTR4			
	PHB2			
	PHF2			
	PHF8			
	PHKA2			
	PI15			
	PI4K2A			
	PIAS2			
	PIGG			
	PIGH			
	PIGM			
	PIGS			
	PIK3R3			
	PIKFYVE			
	PITPNB			
	PIWIL3			
	PKHD1			
	PKP1			
	PLA2G12A			
	PLA2G16			
	PLAC8			
	PLCE1			
	PLCG2			
	PLEKHA3			
	PLEKHA6			
	PLIN5			
	PLXDC1			
	PLXDC2			
	PLXNA1			
	PNP			
	PNPLA3			
	PNPLA8			
	POC1B-GALNT4			
	POFUT1			
	POFUT2			
	POLDIP3			
	POLH			
	POLK			

	POLM		
	POLR1E		
	POLR2E		
	POLR3A		
	POLR3G		
	POMT2		
	POU4F1		
	PPAP2B		
	PPIC		
	PPIL2		
	PPM1K		
	PPM1L		
	PPP1R10		
	PPP1R12B		
	PPP1R15B		
	PPP2CA		
	PPP4R1L		
	PRDM15		
	PRDM16		
	PRELP		
	PRIM1		
	PRKAB2		
	PRKAR2A		
	PRKCB		
	PRKCE		
	PRKG1		
	PRND		
	PROX1		
	PRPF4		
	PRR13		
	PRR15L		
	PRRG3		
	PRRG4		
	PSEN1		
	PSMA4		
	PSMB2		
	PSMD12		
	PSTPIP2		
	PTBP2		
	PTCD1		
	PTCD3		
	PTEN		
	PTGER4		

	PTGFR		
	PTGIS		
	PTGR2		
	PTK6		
	PTP4A1		
	PTPN11		
	PTPRD		
	PTPRG		
	PTRF		
	PURA		
	PVR		
	PVRL1		
	PXMP4		
	PYCARD		
	QPCTL		
	QSOX1		
	RAB11A		
	RAB11FIP1		
	RAB11FIP4		
	RAB13		
	RAB27A		
	RAB2A		
	RAB2B		
	RAB31		
	RAB32		
	RAB3B		
	RAB3IP		
	RAB7L1		
	RABEP1		
	RABGAP1L		
	RABL5		
	RAD1		
	RAD51L3-RFFL		
	RAD52		
	RAD54B		
	RAD9B		
	RAET1L		
	RAP1GDS1		
	RAPGEF2		
	RAPGEFL1		
	RASGRF1		
	RB1CC1		
	RBAK		

	RBBP4			
	RBBP9			
	RBL1			
	RBM12B			
	RBM22			
	RBM41			
	RBM48			
	RBM8A			
	RBMS2			
	RBP2			
	RCAN3			
	RCL1			
	RDH13			
	RDX			
	REEP3			
	REL			
	RELA			
	REST			
	RFESD			
	RFFL			
	RFNG			
	RFX3			
	RGAG4			
	RGP1			
	RGS17			
	RGS9BP			
	RHBDD1			
	RHCG			
	RHOJ			
	RIF1			
	RILPL1			
	RIOK3			
	RIT1			
	RNASEH1			
	RND3			
	RNF115			
	RNF125			
	RNF141			
	RNF149			
	RNF152			
	RNF169			
	RNF222			
	RNF24			

	RNMTL1<br>RORB<br>RP11-111M22.2<br>RP11-156E8.1<br>RP11-192H23.4<br>RP11-302B13.5<br>RP11-343C2.12<br>RP11-766F14.2<br>RPL10A<br>RPL13<br>RPL13A<br>RPL24<br>RPL27A<br>RPL28<br>RPL37A<br>RPL5<br>RPL7L1<br>RPS15A<br>RPS23<br>RPS24<br>RPS6KA1<br>RPS6KA3<br>RRP15<br>RRP1B<br>RTF1<br>RUNDC1<br>RWDD1<br>RYBP<br>SAMD1<br>SAMD10<br>SAMD5<br>SAMHD1<br>SAP18<br>SAPCD2<br>SAR1B<br>SART1<br>SASH1<br>SBK1<br>SBNO1<br>SC5D<br>SCD5<br>SCIMP<br>SCO1		

	SCRG1			
	SCUBE3			
	SCYL2			
	SDC2			
	SEC16B			
	SEC23A			
	SEC23B			
	SEC24D			
	SELRC1			
	SEMA3G			
	SEMA4F			
	SENP1			
	SEPHS1			
	SEPSECS			
	SEPT11			
	SEPT3			
	SERINC1			
	SERPING1			
	SERTAD4			
	SETBP1			
	SETD5			
	SETD7			
	SETDB2			
	SF3B1			
	SF3B3			
	SF3B5			
	SFT2D2			
	SGK494			
	SGTB			
	SH3D19			
	SH3GLB1			
	SH3PXD2A			
	SHCBP1			
	SHROOM2			
	SIGLEC6			
	SIGLEC9			
	SIK1			
	SIK2			
	SIKE1			
	SIRPA			
	SIRPB2			
	SIRT3			
	SLAMF1			

	SLAMF7			
	SLC10A6			
	SLC16A1			
	SLC16A12			
	SLC16A14			
	SLC16A5			
	SLC1A5			
	SLC22A1			
	SLC25A10			
	SLC25A15			
	SLC25A16			
	SLC25A32			
	SLC25A45			
	SLC25A53			
	SLC27A4			
	SLC29A2			
	SLC2A12			
	SLC2A2			
	SLC2A3			
	SLC2A5			
	SLC30A9			
	SLC31A1			
	SLC33A1			
	SLC35B3			
	SLC35D1			
	SLC35E1			
	SLC35E3			
	SLC43A2			
	SLC44A1			
	SLC45A4			
	SLC46A1			
	SLC6A4			
	SLC6A5			
	SLC7A1			
	SLC7A14			
	SLC7A6			
	SLC8A1			
	SLCO1A2			
	SLCO5A1			
	SLFN12L			
	SLFN13			
	SLFN5			
	SLITRK3			

	SLK<br>SLMAP<br>SMAD5<br>SMAD9<br>SMEK2<br>SMIM12<br>SMIM14<br>SMIM15<br>SMIM7<br>SMPD3<br>SMU1<br>SMURF2<br>SNAP47<br>SNN<br>SNRK<br>SNRPD1<br>SNRPD3<br>SNX11<br>SNX2<br>SNX22<br>SNX24<br>SNX27<br>SNX8<br>SOAT1<br>SOCS4<br>SOCS7<br>SORD<br>SOS1<br>SOWAHC<br>SOX1<br>SOX4<br>SP110<br>SP140L<br>SP3<br>SP8<br>SPACA4<br>SPARC<br>SPATA12<br>SPATA13<br>SPATA5<br>SPCS1<br>SPECC1<br>SPEF2		

	SPEM1			
	SPIB			
	SPIRE2			
	SPN			
	SPRTN			
	SPRY3			
	SPRY4			
	SPRYD3			
	SPTBN1			
	SPTBN2			
	SRFBP1			
	SRGAP2			
	SRRD			
	SRSF10			
	SS18L1			
	SSBP2			
	SSH2			
	SSR1			
	ST13			
	ST3GAL1			
	ST6GAL2			
	ST7L			
	STAMBP			
	STARD4			
	STARD5			
	STAT3			
	STAU2			
	STC2			
	STK17B			
	STK25			
	STK35			
	STK38L			
	STOML1			
	STON2			
	STRN3			
	STT3A			
	STX16			
	STX17			
	STX3			
	STX4			
	STXBP5L			
	SUGP1			
	SUN1			

	SURF2			
	SUSD1			
	SUV39H2			
	SUV420H1			
	SUV420H2			
	SVIL			
	SWAP70			
	SYAP1			
	SYDE2			
	SYK			
	SYNJ2BP			
	SYNRG			
	SYS1			
	SYT14			
	SYT7			
	TAB1			
	TACR2			
	TADA2B			
	TAF11			
	TAF13			
	TAF1B			
	TAL1			
	TAL2			
	TANC1			
	TBC1D15			
	TBC1D16			
	TBC1D30			
	TBC1D32			
	TBCEL			
	TBRG4			
	TCEANC2			
	TCEB1			
	TCEB3			
	TCF12			
	TCN2			
	TEAD1			
	TERF2			
	TET1			
	TET2			
	TET3			
	TFCP2			
	TFCP2L1			
	TFEC			

	TG			
	TGS1			
	THAP1			
	THAP2			
	THAP5			
	THAP6			
	THAP8			
	THG1L			
	THOC5			
	THUMPD3			
	TIAF1			
	TIAM1			
	TIFAB			
	TIGD5			
	TIGD6			
	TIMD4			
	TIMM17A			
	TIMM8A			
	TIMM8B			
	TIMP4			
	TIRAP			
	TLCD2			
	TLL1			
	TLR3			
	TLR4			
	TM4SF5			
	TMED5			
	TMED8			
	TMEM127			
	TMEM132D			
	TMEM134			
	TMEM136			
	TMEM141			
	TMEM150C			
	TMEM154			
	TMEM167A			
	TMEM168			
	TMEM176A			
	TMEM181			
	TMEM183A			
	TMEM19			
	TMEM192			
	TMEM199			

	TMEM213			
	TMEM234			
	TMEM236			
	TMEM241			
	TMEM251			
	TMEM26			
	TMEM33			
	TMEM41A			
	TMEM44			
	TMEM67			
	TMEM86A			
	TMOD2			
	TMOD3			
	TMPRSS4			
	TMUB2			
	TNFAIP1			
	TNFRSF10A			
	TNFRSF10B			
	TNFRSF13C			
	TNFRSF9			
	TNFSF15			
	TNFSF8			
	TNR			
	TNRC18			
	TOB2			
	TOMM40			
	TOMM7			
	TOMM70A			
	TP53INP1			
	TP53INP2			
	TP53RK			
	TP53TG5			
	TPBG			
	TPD52L3			
	TPST2			
	TRABD2A			
	TRABD2B			
	TRAF1			
	TRAF4			
	TRAFD1			
	TRAM2			
	TRAPPC2L			
	TRIM24			

	TRIM33<br>TRIM44<br>TRIM45<br>TRIM58<br>TRIM66<br>TRIM71<br>TRIM72<br>TRIP11<br>TRPM7<br>TRPS1<br>TRPV2<br>TRPV3<br>TRPV5<br>TRUB2<br>TSEN2<br>TSKU<br>TSNARE1<br>TSPAN4<br>TSPAN5<br>TTC26<br>TTC28<br>TTC31<br>TTC38<br>TTC39A<br>TTC39B<br>TTL<br>TTLL3<br>TTLL7<br>TTPA<br>TUB<br>TVP23C<br>TXK<br>TXNDC15<br>TYRP1<br>TYW3<br>TYW5<br>U2SURP<br>UACA<br>UBA2<br>UBA6<br>UBC<br>UBE2B<br>UBE2D4				

	UBE2N			
	UBE2Q1			
	UBE2W			
	UBIAD1			
	UBN1			
	UBR3			
	UBTD2			
	UCHL3			
	UGGT1			
	UHRF1BP1			
	ULBP2			
	ULK1			
	ULK3			
	UMPS			
	UNK			
	UNKL			
	UPF3A			
	UPK1B			
	UQCC1			
	UQCR11			
	URB1			
	URGCP			
	USP13			
	USP15			
	USP34			
	USP40			
	USP42			
	USP46			
	USP49			
	USP54			
	USP6NL			
	USP9X			
	UTP15			
	UTP6			
	VAPA			
	VAPB			
	VASH2			
	VCPKMT			
	VEGFA			
	VEZT			
	VHL			
	VPS13D			
	VPS35			

	VPS41		
	VSNL1		
	VSTM4		
	VTA1		
	VTI1A		
	VWA2		
	VWC2		
	WDFY1		
	WDPCP		
	WDR12		
	WDR17		
	WDR20		
	WDR26		
	WDR52		
	WDR53		
	WDR5B		
	WDR70		
	WDR91		
	WDTC1		
	WEE1		
	WHAMM		
	WHSC1		
	WIPF2		
	WNT7B		
	WNT8B		
	WRN		
	WSB1		
	WSB2		
	WT1		
	WWC1		
	XAF1		
	XDH		
	XIAP		
	XKR6		
	XKR7		
	XRCC5		
	XRN1		
	XXYLT1		
	YAE1D1		
	YIPF7		
	YME1L1		
	YOD1		
	YPEL1		

	YPEL2			
	YTHDF3			
	YWHAZ			
	YY1			
	Z98049.1			
	ZBED1			
	ZBED3			
	ZBTB21			
	ZBTB34			
	ZBTB37			
	ZBTB43			
	ZBTB44			
	ZBTB5			
	ZBTB6			
	ZBTB8A			
	ZBTB8B			
	ZC3H8			
	ZCCHC14			
	ZCCHC4			
	ZDHHC20			
	ZDHHC21			
	ZDHHC3			
	ZDHHC9			
	ZFAND4			
	ZFP30			
	ZFP36L2			
	ZFP90			
	ZFP91			
	ZFPM1			
	ZFR2			
	ZFX			
	ZFYVE20			
	ZFYVE26			
	ZHX3			
	ZKSCAN1			
	ZKSCAN3			
	ZKSCAN4			
	ZKSCAN8			
	ZMIZ2			
	ZNF106			
	ZNF107			
	ZNF12			
	ZNF132			

	ZNF148		
	ZNF154		
	ZNF160		
	ZNF174		
	ZNF177		
	ZNF189		
	ZNF225		
	ZNF229		
	ZNF230		
	ZNF234		
	ZNF250		
	ZNF264		
	ZNF276		
	ZNF28		
	ZNF281		
	ZNF285		
	ZNF292		
	ZNF317		
	ZNF320		
	ZNF321P		
	ZNF324B		
	ZNF326		
	ZNF346		
	ZNF354B		
	ZNF362		
	ZNF366		
	ZNF37A		
	ZNF383		
	ZNF394		
	ZNF41		
	ZNF436		
	ZNF44		
	ZNF445		
	ZNF468		
	ZNF486		
	ZNF488		
	ZNF491		
	ZNF496		
	ZNF512B		
	ZNF525		
	ZNF527		
	ZNF532		
	ZNF543		

	ZNF546<br>ZNF555<br>ZNF557<br>ZNF570<br>ZNF573<br>ZNF577<br>ZNF578<br>ZNF584<br>ZNF594<br>ZNF607<br>ZNF616<br>ZNF620<br>ZNF626<br>ZNF639<br>ZNF641<br>ZNF652<br>ZNF660<br>ZNF667<br>ZNF669<br>ZNF674<br>ZNF677<br>ZNF695<br>ZNF697<br>ZNF699<br>ZNF70<br>ZNF701<br>ZNF71<br>ZNF716<br>ZNF724P<br>ZNF726<br>ZNF730<br>ZNF736<br>ZNF737<br>ZNF74<br>ZNF770<br>ZNF776<br>ZNF780B<br>ZNF783<br>ZNF786<br>ZNF790<br>ZNF791<br>ZNF793<br>ZNF8			

	ZNF813			
	ZNF827			
	ZNF843			
	ZNF845			
	ZNF850			
	ZNF852			
	ZNF862			
	ZNF878			
	ZNHIT6			
	ZNRF3			
	ZRANB1			
	ZSCAN2			

Supplementary file 2: Genes targeted by groups of miRs. Filtered targets were found after the removal of targets shared between groups of miRs that led to opposite effects (G1a vs G2 and G1b vs G3).
