# Supplementary material for: A High-Content Screening Approach to Identify MicroRNAs Against Head and Neck Cancer Cell Survival and EMT in an Inflammatory Microenvironment
Source: Front Oncol. 2019 Nov 8;9:1100. doi: 10.3389/fonc.2019.01100 (PMC6856008; doi:10.3389/fonc.2019.01100)
Supplement: Supplementary file 3 [file Data_Sheet_3.pdf]

Supplementary file 3: Signaling pathways enriched with filtered targets from groups of miRs.

G1a

Term	PValue	Fold Enrichment	FDR
hsa05410:Hypertrophic cardiomyopathy (HCM)	0.014	3.09	16.62
hsa04510:Focal adhesion	0.018	2.05	21.12
hsa05414:Dilated cardiomyopathy	0.020	2.87	23.28
hsa05412:Arrhythmogenic right ventricular cardiomyopathy (ARVC)	0.029	2.97	31.53
hsa05200:Pathways in cancer	0.035	1.61	36.40
hsa04550:Signaling pathways regulating pluripotency of stem cells	0.042	2.16	41.95
hsa04750:Inflammatory mediator regulation of TRP channels	0.043	2.46	42.80
hsa04015:Rap1 signaling pathway	0.044	1.87	44.03
hsa04340:Hedgehog signaling pathway	0.058	4.47	53.50
hsa04360:Axon guidance	0.058	2.14	53.75
hsa04911:Insulin secretion	0.061	2.48	55.47
hsa04014:Ras signaling pathway	0.069	1.74	60.29
hsa04068:FoxO signaling pathway	0.075	2.03	63.15
hsa03015:mRNA surveillance pathway	0.079	2.32	65.42
hsa04724:Glutamatergic synapse	0.082	2.12	66.72
hsa00564:Glycerophospholipid metabolism	0.093	2.22	71.50
hsa04270:Vascular smooth muscle contraction	0.098	2.03	73.31

G1b

Term	PValue	Fold Enrichment	FDR
hsa04930:Type II diabetes mellitus	0.002	6.80	2.10
hsa05212:Pancreatic cancer	0.006	5.02	7.72
hsa04917:Prolactin signaling pathway	0.009	4.60	10.98
hsa05220:Chronic myeloid leukemia	0.010	4.53	11.60
hsa04114:Oocyte meiosis	0.014	3.49	16.49
hsa05161:Hepatitis B	0.016	3.00	18.68
hsa04014:Ras signaling pathway	0.022	2.41	24.17
hsa04723:Retrograde endocannabinoid signaling	0.037	3.23	37.36
hsa04910:Insulin signaling pathway	0.040	2.76	39.82
hsa04668:TNF signaling pathway	0.044	3.08	42.85
hsa04725:Cholinergic synapse	0.052	2.94	48.42
hsa04727:GABAergic synapse	0.069	3.20	59.07
hsa05222:Small cell lung cancer	0.069	3.20	59.07
hsa04914:Progesterone-mediated oocyte maturation	0.074	3.13	61.64
hsa05215:Prostate cancer	0.076	3.09	62.90
hsa05223:Non-small cell lung cancer	0.082	3.89	65.49
hsa04150:mTOR signaling pathway	0.089	3.75	68.61
hsa04380:Osteoclast differentiation	0.091	2.49	69.44
hsa04010:MAPK signaling pathway	0.094	1.92	70.62
hsa04068:FoxO signaling pathway	0.098	2.44	72.20

G2			
Term	PValue	Fold Enrichment	FDR
hsa05161:Hepatitis B	0.000	2.08	0.39
hsa05222:Small cell lung cancer	0.001	2.41	0.82
hsa05200:Pathways in cancer	0.002	1.51	2.25
hsa05205:Proteoglycans in cancer	0.003	1.73	3.53
hsa04210:Apoptosis	0.004	2.44	4.86
hsa04931:Insulin resistance	0.004	2.00	5.63
hsa04550:Signaling pathways regulating pluripotency of stem cells	0.004	1.85	5.73
hsa04917:Prolactin signaling pathway	0.005	2.28	6.21
hsa04070:Phosphatidylinositol signaling system	0.008	1.98	9.91
hsa05169:Epstein-Barr virus infection	0.009	1.65	10.76
hsa05164:Influenza A	0.009	1.68	11.61
hsa04920:Adipocytokine signaling pathway	0.011	2.16	13.42
hsa04068:FoxO signaling pathway	0.011	1.77	13.54
hsa05145:Toxoplasmosis	0.011	1.83	14.09
hsa05215:Prostate cancer	0.014	1.96	17.18
hsa00512:Mucin type O-Glycan biosynthesis	0.021	2.79	24.01
hsa04662:B cell receptor signaling pathway	0.023	2.03	26.48
hsa04923:Regulation of lipolysis in adipocytes	0.031	2.12	33.78
hsa04620:Toll-like receptor signaling pathway	0.033	1.73	36.19
hsa04066:HIF-1 signaling pathway	0.035	1.76	37.27
hsa03040:Spliceosome	0.036	1.62	38.61
hsa04932:Non-alcoholic fatty liver disease (NAFLD)	0.037	1.57	39.39
hsa04020:Calcium signaling pathway	0.040	1.51	41.52
hsa04350:TGF-beta signaling pathway	0.044	1.80	44.56
hsa05231:Choline metabolism in cancer	0.044	1.71	44.62
hsa04010:MAPK signaling pathway	0.045	1.40	45.47
hsa04664:Fc epsilon RI signaling pathway	0.046	1.91	46.38
hsa04310:Wnt signaling pathway	0.050	1.56	49.22
hsa04022:cGMP-PKG signaling pathway	0.053	1.50	51.25
hsa05142:Chagas disease (American trypanosomiasis)	0.054	1.66	52.22
hsa00230:Purine metabolism	0.056	1.47	52.96
hsa04064:NF-kappa B signaling pathway	0.056	1.74	52.96
hsa05134:Legionellosis	0.058	2.00	54.26
hsa00562:Inositol phosphate metabolism	0.060	1.82	55.85
hsa00514:Other types of O-glycan biosynthesis	0.061	2.44	56.51
hsa04750:Inflammatory mediator regulation of TRP channels	0.066	1.65	59.31
hsa04922:Glucagon signaling pathway	0.071	1.64	61.91
hsa05214:Glioma	0.074	1.83	63.60
hsa04151:PI3K-Akt signaling pathway	0.077	1.28	65.35
hsa05168:Herpes simplex infection	0.079	1.42	66.18
hsa04728:Dopaminergic synapse	0.080	1.52	66.92
hsa01100:Metabolic pathways	0.082	1.13	67.52
hsa05152:Tuberculosis	0.092	1.40	72.17
hsa04380:Osteoclast differentiation	0.095	1.48	73.18
hsa04510:Focal adhesion	0.095	1.36	73.19
hsa04014:Ras signaling pathway	0.098	1.34	74.55

G3			
**Term**	**PValue**	**Fold Enrichment**	**FDR**
hsa04068:FoxO signaling pathway	0.001	2.52	1.34
hsa04150:mTOR signaling pathway	0.008	3.08	9.82
hsa05205:Proteoglycans in cancer	0.023	1.79	25.99
hsa04010:MAPK signaling pathway	0.031	1.64	33.62
hsa04931:Insulin resistance	0.044	2.02	44.32
hsa04921:Oxytocin signaling pathway	0.054	1.76	51.21
hsa04932:Non-alcoholic fatty liver disease (NAFLD)	0.077	1.71	64.48
hsa05133:Pertussis	0.081	2.12	66.79
hsa04142:Lysosome	0.082	1.81	67.12
hsa04210:Apoptosis	0.090	2.24	70.44

Signaling pathways enriched with filtered targets from groups of miRs. With aid of the Database for Annotation, Visualization and Integrated Discovery (DAVID, version 6.7), we found the signaling pathways enriched with filtered targets from groups of miRs.
